# The Endocannabinoid System: A Target for Cancer Treatment

**DOI:** 10.3390/ijms21030747

**Published:** 2020-01-23

**Authors:** Chiara Laezza, Cristina Pagano, Giovanna Navarra, Olga Pastorino, Maria Chiara Proto, Donatella Fiore, Chiara Piscopo, Patrizia Gazzerro, Maurizio Bifulco

**Affiliations:** 1 Institute of Endocrinology and Experimental Oncology, IEOS CNR, 80131 Naples, Italy; 2 Department of Molecular Medicine and Medical Biotechnology, University of Naples “Federico II”, 80131 Naples, Italy; pagano.cris@gmail.com (C.P.); vanna.navarra@libero.it (G.N.); olga.pastorino@gmail.com (O.P.); 3 Department of Pharmacy, University of Salerno, 84084 Fisciano (SA), Italy; maproto@unisa.it (M.C.P.); dfiore@unisa.it (D.F.); cpiscopo@unisa.it (C.P.);

**Keywords:** Cannabinoids, metastasis, cancer stem cell, angiogenesis

## Abstract

In recent years, the endocannabinoid system has received great interest as a potential therapeutic target in numerous pathological conditions. Cannabinoids have shown an anticancer potential by modulating several pathways involved in cell growth, differentiation, migration, and angiogenesis. However, the therapeutic efficacy of cannabinoids is limited to the treatment of chemotherapy-induced symptoms or cancer pain, but their use as anticancer drugs in chemotherapeutic protocols requires further investigation. In this paper, we reviewed the role of cannabinoids in the modulation of signaling mechanisms implicated in tumor progression.

## 1. Introduction

Cannabinoids are comprised of a group of chemical compounds found in the marijuana plant *Cannabis sativa* which produces more than 500 different compounds throughout its life cycle, of which more than 100 are identified as phytocannabinoids. The two major components are delta-9-tetrahydrocannabinol (Δ9-THC) and cannabidiol (CBD). Other minor components are cannabinol (CBN), tetrahydrocannabivarin (THCV) and cannabigerol (CNG). The Δ9-THC is the psychoactive cannabinoid that binds to CB1 and CB2 cannabinoid receptors identified in mammalian organisms. CBD does not have psychotropic activity, and is used to treat neurological diseases and cancer. CBD, unlike Δ9-THC, has lower CB1 and CB2 receptor affinity and it is an inverse agonist at the human CB2 receptor [1,2]. The Δ9-THC executes several biological effects that mimic those of endogenous substances through the activation of specific cannabinoid receptors. These substances are named endocannabinoids. The two major endocannabinoids are N-arachidonoyl-ethanolamine (AEA) and 2-arachidonoylglycerol (2-AG) synthesized from arachidonic acid. The CB1 and CB2 receptors, the endocannabinoids and the biochemical machinery to produce and to degrade these lipids, are known as the endocannabinoid system (ECS). It plays an important role in the organism’s physiology. Dysregulation of the endocannabinoid system, owing to variation in the expression and function of cannabinoid receptors or enzymes or the concentration of endocannabinoids, has been associated with several diseases, such as neurodegenerative disorders, multiple sclerosis, inflammation, epilepsy, schizophrenia, glaucoma, cardiovascular diseases, obesity and cancer [3,4]. In recent years, further components have expanded this original definition of the endocannabinoid system. These components comprise newly discovered endogenous cannabinoid receptor ligands such as 2-arachidonoyl glyceryl ether (noladin ether, 2-AGE), O-arachidonoylethanolamine (virodhamine), N-arachidonoyldopamine (NADA) and oleic acid amide (oleamide, OA) as well as further receptor targets such as G protein-coupled receptor GPR55 and peroxisome proliferator-activated receptors (PPARs) [5]. However, it is known that other receptors participate in cannabinoid signaling. Recently, it has been discovered that cannabinoids can affect a subset of transient receptor potential (TRP) channels. The TRP vanilloid (TRPV), TRP ankyrin (TRPA) and TRP melastatin (TRPM) subfamilies were all found to contain channels that can be modulated by several endogenous, phytogenic, and synthetic cannabinoids. Six TRP channels from the three subfamilies mentioned above have been reported to mediate cannabinoid activity: TRPV1, TRPV2, TRPV3, TRPV4, TRPA1 and TRPM8. Although CB1 and CB2 are considered to be the canonical cannabinoid receptors, there is significant overlap between cannabinoids and ligands of TRP receptors. The first endogenous agonist of TRPV1 was the endocannabinoid, anandamide (AEA). Similarly, N-arachidonyl dopamine (NADA) and AEA were the first endogenous TRPM8 antagonists discovered [6]. Besides receptors, ECS encompasses several enzymes that regulate biosynthesis and degradation of endocannabinoids, which potentially represent an indirect pharmacological target. The fatty acid amide hydrolase (FAAH) is the catabolic enzyme mainly for AEA degradation and, with lower affinity, of oleoylethanolamide (OEA) and palmitoylethanolamide (PEA), while the major enzyme responsible for 2-AG degradation is monoacylglycerol lipase (MAGL). Interestingly, FAAH and MAGL expression were found upregulated in cancer tissues [7,8]. Other enzymes, like lysosomal hydrolase N-acylethanolamine hydrolyzing acid amidase (NAAA) that degrades AEA, OEA, and PEA, constitute potential targets in cancer [5].

## 2. Anticancer Effects of Cannabinoids

**Figure 1:**
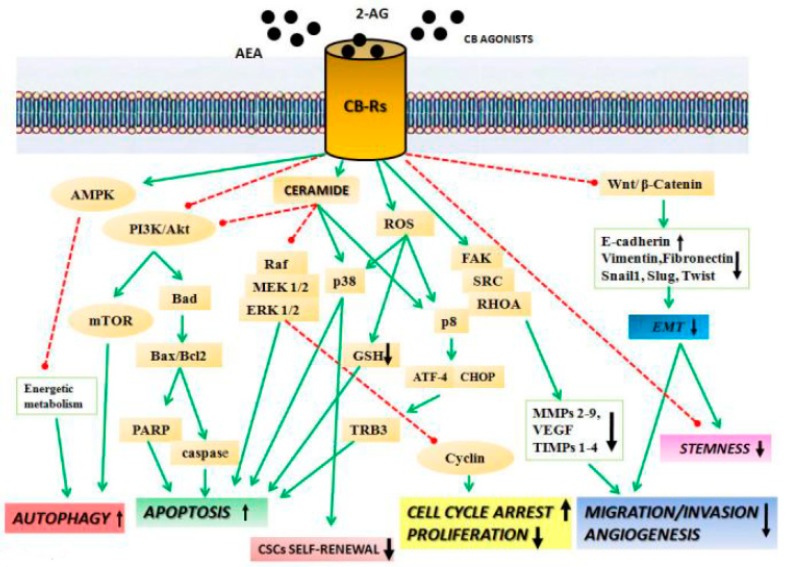
Schematic representation of the main anticancer molecular mechanisms mediated by cannabinoid receptors’ activation “↑, upregulation” and “↓, downregulation”. Cannabinoid receptor (CB-R) agonists inhibit cancer cell proliferation through various receptor-mediated mechanisms. CB-R agonist induces cancer cell death via apoptosis, mediated by the activation of different transcription factors (proapoptotic Bcl2 family transcription factor and mitogen-activated protein kinase (MAPK) pathway) and de novo synthesis of ceramide and reactive oxygen species (ROS) production. CBs block cancer cells’ proliferation by inhibiting extracellular signal regulated kinase (ERK) signaling. They also reduce cell migration and angiogenesis, inhibiting the focal adhesion kinase/proto-oncogene tyrosine-protein kinase Src/transforming protein RhoA (FAK/SRC/RhoA) pathway. CBs prevent cancer epithelial mesenchymal transition (EMT), inhibiting Wnt/β-catenin pathway, and induce autophagy by activation of mammalian target of rapamycin (mTOR) and AMP-activated protein kinase (AMPK) pathways. CBs can impair stemness and cancer stem cells’ (CSCs) self-renewal. (Akt (protein kinase B), PI3K (phosphoinositol-3-kinase) Raf (serine/threonine-protein kinase)).

Considering the high complexity of ECS and the distribution of its components, it is likely that (endo)cannabinoids potentially impact a multitude of cancer-related signaling pathways. Both CB1 and CB2 are seven-transmembrane domain receptors coupled to Gi/o protein. Their activation triggers several pathways widely involved in cancer. Mainly, antiproliferative and pro-apoptotic effects were attributed to activation of the alpha subunit of Gi/o that leads to inhibition of adenylate cyclase and, in turn, of cyclic Adenosine Monophosphate (cAMP) synthesis and protein kinase A (PKA) activity, with consequent downregulation of gene transcription [9]. On the other hand, antiproliferative and pro-apoptotic effects of both CB1 and CB2 agonists have been attributed also to their ability to increase the synthesis of the pro-apoptotic sphingolipid ceramide. In leukemic cells, ceramide can induce apoptosis by regulation of p38 MAPK (mitogen-activated protein kinase) signaling, while in glioma cells it up-regulates the endoplasmic reticulum (ER) stress-related gene, those encoding the transcription factors activating transcription factor 4 (ATF-4) and C/EBP homologous protein (CHOP), and the stress-related pseudokinase [7] (Figure 1). In lung cancer, the ceramide-dependent pro-apoptotic effect triggered by AEA and CBD seems to be mediated by an up-regulation of cyclooxygenase 2 (Cox-2) expression and by the increased synthesis of the pro-apoptotic prostaglandin E-2 (PGE2) [8]. Interestingly, it was reported that the CB receptor agonist Δ9-THC promotes autophagy-mediated apoptosis through the upregulation of Tribbles homolog 3 (TRB3), which leads to inhibition of the protein kinase B/ the target of rapamycin kinase complex 1 (Akt/mTORC1)1 axis. Furthermore, Akt inhibition determines the activation of the pro-apoptotic protein BAD (a Bcl2-associated agonist of cell death) [5,8]. Other reports suggested that cannabinoid-induced autophagy occurs through activation of calcium/calmodulin-dependent protein kinase β (CAMKKβ), which subsequently phosphorylates AMP-activated kinase (AMPK) [10]. In cancer cells, the inhibition of Akt has been directly linked to cannabinoid’s ability to control of cell cycle checkpoints. Specifically, in a CB1-dependent manner, AEA (or its synthetic analogs Met-F-AEA) induces a cell cycle arrest at the G1-S transition through up-regulation of p21^waf^, p27^kip1^ and cell division cycle 25A Cdc25A proteolysis and inhibition of the cyclin E–Cdk2 kinase complex [7,8]. However, other authors reported that Δ9-THC upregulating p21^waf^ suppresses cell division cycle 2 Cdc2–cyclin B activation and induces a G2/-M cell cycle arrest. Consequently, a reduction of Rb activity has been verified [7,8] (Figure 1).

### 2.1. Cannabinoids Inhibit Migration, Invasion, and Angiogenesis

An increasing number of reports highlighted the role of cannabinoids in cancer spreading, specifically in invasion, angiogenesis, migration, and metastasis mechanism [5,6,7,8,9,10]. Likely, these mechanisms can be triggered through the modulation of different pathways. Activation of Gi/o upon binding of agonists (e.g., AEA, 2-AG, Δ9-THC, [(3R)-2,3-dihydro-5-methyl-3-(4-morpholinylmethyl)pyrrolo[1,2,3-de]-1,4-benzoxazin-6-yl]-1-naphthalenyl-methanone, monomethanesulfonate (WIN-55,212-2)) on CB1 and CB2 receptors inhibits the RHOA (ras homolog gene family, member A)- focal adhesion kinase - Proto-oncogene tyrosine-protein kinase Src (RhoA-FAK-Src) axis. As a consequence, a down-regulation of the proangiogenic factors vascular endothelial growth factor (VEGF), placental growth factor (PlGF), and angiopoietin-2 (Ang-2) occurs [8]. Moreover, cannabinoids inhibit angiogenesis and invasion, inducing the release of tissue inhibitor of matrix metalloproteinases-1 (TIMP-1) that, in turn, acts as an endogenous inhibitor of matrix metalloproteinase 2 (MMP2) [8]. Another interesting observation derives from glioma and breast cancer, where the treatment with CBD or the CB2 agonist O-1663 exerts the inhibitory effect on cancer cell invasion through a down-regulation of Id-1 and Sox-2 protein expression [6]. Enforcing the antitumor potential of cannabinoids, some results suggested their ability to affect epithelial mesenchymal transition (EMT) and chemoresistance. In breast cancer cells, 2-methyl-2′-F-anandamide (Met-F-AEA) inhibits Wnt/β-catenin pathway through a reduction of β-catenin nuclear translocation and transcriptional activity, which culminates with a downregulation of β-catenin target genes, such MMP2, c-Myc, and cyclin D. The inhibition of the Wnt pathway was accompanied by a reduction of mesenchymal markers (e.g., vimentin, N-cadherin, Snail, and Slug) [11] (Figure 1). Further, the compound JZL184, a potent selective inhibitor of MAGL enzyme responsible for degrading the endocannabinoid (2-AG), was able to regulate the EMT process, reducing EMT markers and upregulating epithelial markers such E-cadherin [12]. Other reports emphasized the role of cannabinoids and, in particular, of CBD and Δ9-THC in chemoresistance mechanisms, highlighting their potential use in combined therapy with several chemotherapeutic agents [8]. Interestingly, the improvement of cancer cells’ response to chemotherapeutic agents seems to be mainly ascribable to a decreased p42/44 MAPK activity and to the inhibition of P-glycoprotein and ATP (adenosine triphosphate)-binding cassette super-family G member 2 (ABCG2) [13]. An appealing pharmacologic approach for ECS targeting derives from the possibility of an indirect strategy. Inhibition of enzymes such MAGL, FAAH, and NAAA prevents the degradation of (endo)cannabinoids, increasing their availability on CB receptors, thus recapitulating the direct effect of agonists. The direct involvement in cancer of enzymes involved in synthesis and degradation of the endogenous ligands has been confirmed by several studies that reported the up-regulated expression of NAAA, MAGL, and FAAH in different cancers [7]. The N-cyclohexanecarbonylpentadecylamine and other new synthetic NAAA inhibitors were found to induce cell death of neuroblastoma and bladder cancer cells [14,15]. Encouraging results were obtained in regards to the FAAH inhibitors, AA-5HT (N-arachidonoyl-serotonin) and URB597. Used alone, AA-5HT exerts an antiproliferative effect in glioma and thyroid cancer [5]. Moreover, in azoxymethane (AOM)-induced colon cancer murine models it reduces the onset of aberrant crypt foci, probably in a CB1-dependent manner [16]. URB597 showed a potent antiproliferative effect used in combination with AEA (or Met-F-AEA) in neuroblastoma, lung, and colon cancer, or combination with PEA in melanoma cancer cells [5]. In lung cancer, both AA-5HT and URB597 contrast tumor invasion through TIMP-1 upregulation, likely in a CB2- and TRPV1-dependent way [17]. The MAGL inhibitor JZL184, inhibiting proliferation and tumor cell invasion, induces apoptosis in colon and prostate cancer [18,19]. Interestingly, URB602, another MAGL inhibitor, reduces AOM-induced preneoplastic lesions and reduces tumor volume in vivo in colorectal cancer (CRC) models. Moreover, URB602 inhibits angiogenesis down-regulating vascular endothelial growth factor receptor (VEGFR) and fibroblast growth factor 2 (FGF-2) [20].

### 2.2. Cannabinoids Affect non-CB1/CB2 Receptors

Beyond CB1 and CB2 receptors, it is well known that other non-CB receptors are involved in antitumor action of cannabinoids, representing another interesting target for therapeutic intervention. TRPVs are non-selective cation channels found to be a key player in cannabinoids-induced anticancer effects. TRPV1 and TRPV2 are the best-studied receptors of this family that appear to be up-regulated in several cancers. AEA activates TRPV1 and, Δ9-THC acts on TRPV2, while the non-psychoactive CBD is able to activate both TRPV1 and TRPV2 [6]. The TRPV-dependent pro-apoptotic effect of cannabinoids involves the intracellular calcium (Ca^2+^) influx, which increases upon ligand binding. The Ca^2+^ increase culminates with triggering of several mechanisms such apoptosis (via mitochondrial transmembrane potential alterations), an increase of intracellular reactive oxygen species (ROS), autophagy, cell cycle arrest, or inhibition of cancer cells invasion through up-regulation of TIMP-1 [21]. Some evidence supports the role of PPARs in cannabinoids-mediated antitumor action. The cannabinoids AEA and, WIN-55,212-2 as well as the endocannabinoid-like substances PEA, and OEA activate PPARα, while AEA, R(+)-methanandamide, CBD, and Δ9-THC activate PPARγ [22]. Of note, an indirect up-regulation of PPARγ has been found in lung cancer, where the CBD-mediated proapoptotic and antiproliferative effect occurs via the up-regulation of Cox2 and prostaglandins production, with consequent PPARγ nuclear translocation [23]. Despite the poor sequence similarity with CB receptors, GPR55 has been found as a putative cannabinoid receptor. The endogenous ligand of GPR55 is the phospholipid lysophosphatidylinositol (LPI), but it can be activated by AEA, 2-AG, virodhamine, and PEA. On the other hand, it was reported that both CBD and the synthetic SR141716 (rimonabant) play the role of GPR55 antagonists [8,24,25]. LPI/GPR55 axis creates an autocrine loop, in which LPI-mediated stimulation of GPR55 activates the pro-tumorigenic Akt and extracellular receptor kinase (ERK) pathways. Thus, pharmacological blockade of GPR55 mediated by ligands such as CBD and SR141716 translates in antiproliferative effects [25,26]. Attractive evidence derives from the ability of GPR55 and other receptors to dimerize. Recently, heteromers with cannabinoids receptors emerge as potential targets in cancers. Generally, heteromerization of receptors produces a modification in ligand binding and affinity that sometimes produces a characteristic response, likely disease specific [24]. Among others, the CB2 receptor- GPR55 heteromers were found to be involved in cancer cell fate of different cancers, such as breast cancer, where its targeting reduces tumor growth [27]. Moreover, these heteromers seem to be involved in cancer-related processes, being over-expressed in bones and hematopoietic cells. In breast and prostate cancer cells, C-X-C chemokine receptor type 4 (CXCR4)-CB2 receptor heteromers regulate proliferation, adhesion, and invasion, thus metastatic potential. In this case, cannabinoids agonists of the CB2 receptor inhibit the effect of CXCR4 agonist, thus indirectly affect invasion [28,29]. In breast cancer, both CB2 receptor and human V-Erb-B2 avian erythroblastic leukemia viral oncogene homolog 2 (HER2) were up-regulated, constituting an indisputable hallmark of cancer. The finding of HER2-CB2 receptor heteromers suggested that dual targeting of both receptors in HER2+ breast cancer produces a synergistic antitumor effect [30].

## 3. Gastrointestinal Cancers

The ECS, creating a regulatory network, is involved in both physiological and pathophysiological processes of the gastrointestinal (GI) tract. Here CB1 and CB2 receptors have been detected in the enteric nervous system and epithelial cells [31].

In colorectal cancer (CRC) the expression of ECS components has been found increased and associated with poor prognosis and advanced stage of disease [32]. Recently, it has been reported that the AEA and 2-AG levels, as well as the expression of AEA-synthetizing enzymes and degrading enzymes, has been founded to be higher in CRC than in normal mucosa [33,34]. To the contrary, the down-regulating CB1 receptor expression observed in colon cancer tissues compared to normal mucosa [35,36] might be due to the epigenetic mechanism of DNA hypermethylation at CpG islands around the transcription start site of receptor gene (*CNR1*) [37]. The knockout of CB1 receptor in Apc^Min/+^ mice accelerated intestinal adenoma growth, suggesting a tumor suppressive role of CB1 [36,37]. In these models, CB1 was up-regulated in inflamed non-tumor tissue and down-regulated in tumor lesions, while GPR55 was found to be regulated exactly in an inverse manner, acting oppositely to CB1. Indeed, GPR55^−/−^ mice exhibited an alteration of leucocyte population in the tumor microenvironment and, concomitantly, a reduced expression of pro-tumorigenic factors (e.g., cyclooxygenase 2 (COX-2), signal transducer and activator of transcription 3 (STAT3), and proliferating cell nuclear antigen (PCNA) [36]. In other studies, CB1 up-regulation was associated with a shorter survival time of CRC patients with stage II microsatellite-stable [32] or stage IV tumors [38]; whereas, CB2 receptor up-regulation in CRC tissue was correlated to higher proliferation levels and lymph node involvement, suggesting that also its expression could be a negative prognostic factor [34]. Conversely, in hepatocarcinoma high levels of both receptors have been associated with better disease-free survival rates [39,40]. Despite the reported evidence, the altered expression of CB receptors in several GI cancers is not strictly related to a straightforward cause and effect and must be further investigated. However, cannabinoids’ action in GI cancers has been demonstrated in vitro and in vivo, indicating their antiproliferative, proapoptotic, and antimetastatic properties [41]. Of note, it has been demonstrated that endogenous cannabinoid agonists, such as AEA (0.5–5 μM) and its metabolic-stable analogous, Meth-AEA (0.5–5 μM), diminished the volume and the density of gastric carcinomas cells, inducing apoptosis and necrosis, respectively [42]. The endocannabinoid AEA (10 μM) reduced the growth of cholangiocarcinoma in vivo model, upregulating Wnt5a expression with subsequent activation of receptor tyrosine kinase-like orphan receptor 2 (Ror2) and c-Jun N-terminal kinase JNK [43]. The growth-suppressing effects of AEA (10^−9^ to 10^−5^ M) involved GPR55 activation and subsequent translocation of Fas death receptor into the lipid raft structures [44]. Also, AEA (10 μM) downregulated the expression of angiogenic factors, vascular endothelial growth factor-C (VEGF-C), vascular endothelial growth factor –receptor 2 (VEGF-R2), and vascular endothelial growth factor-receptor 3VEGF-R3 in tumors [43]. We previously showed that the increase of AEA availability obtained either exogenously by the administration of Met-F-AEA (10 μM) or endogenously by selective FAAH inhibition with URB597 (0.1 μM) induced the CB1 expression and reduced the proliferation of CRC cell lines. On the other hand, the selective CB1 antagonist AM-251 at concentration of 3 μM reverted the Met-F-AEA antiproliferative effect, suggesting that the cell growth inhibition could be due to CB1 activation. Results demonstrated that the control on CRC cell line proliferation was mediated by increased expression of CB1 receptor through transcriptional activation of the *CNR1* promoter. Furthermore, *CNR1* gene behaves as a typical steroid-regulated target, suggesting a fine link between the endocannabinoid system and steroids in CRC [45]. Interestingly, pyrrolo-1,5-benzoxazepine-15 (PBOX-15) (from 0.001 to 5 µM), a synthetic inhibitor of FAAH, demonstrated a strong antiproliferative and proapoptotic effect in CRC cell lines. Moreover, it was observed that nanomolar concentration of PBOX-15 increased the anticancer action of 5-fluouracil (5-FU) [46].

In the gastric cell line, SGC-7901, the natural compound CBD, at the concentrations ranging from 10 μg/mL to 40 μg/mL, induced G0/G1 cell cycle arrest by decreasing the expression of cyclin-dependent kinase 2/ cyclin E (CDK2/cyclin E) and upregulating ataxia telangiectasia mutated (ATM) levels, thus activating the mitochondrial-dependent apoptotic pathway [47]. Kargl et al. provided evidence that the GPR55-lysophosphatidylinositol axis is crucial in CRC progression. They found that CBD (2.5 μM), antagonizing GPR55, was able to reduce adhesion and migration of HCT116 to a HUVEC cell monolayer in vitro and liver metastases in vivo [48]. At noncytotoxic concentrations, CBD (10 μM) exerts antiproliferative effects in CRC models through multiple mechanisms. In vitro studies suggested that CBD protected DNA from oxidative damage, increased endocannabinoid levels, and reduced cell proliferation through different mechanisms, involving CB1, TRPV1, and PPARγ [49]. The chemopreventive effect of CBD was also verified in vivo, in experimental models of chemically induced colon carcinogenesis. More specifically, CBD, at the dose of 1 mg/kg, reduced aberrant crypt foci (ACF) formation and the number of polyps and tumors in azoxymethane (AOM)-treated mice. The authors found that CBD counteracted AOM-induced upregulation of the phosphorylated form of Akt protein [49]. More recently, the pro-apoptotic effect of CBD in CRC cells has been ascribable to the excessive ROS production by mitochondria, ER stress induction, and Noxa activation [50]. Additionally, CBD (1 mg/kg) effect has been investigated in a murine model of CT26 cell line-induced colon cancer. Results showed that anti-angiogenetic and antimetastatic effects of CBD were associated with VEGF downregulation. Moreover, CBD reduced interleukin 6 (IL-6) and interleukin 8 (IL-8) serum levels of the treated group with respect to control group [51]. Greenhough et al. demonstrated that Δ9-THC (2.5 μM), via CB1 activation, induced apoptosis through inhibition of phosphoinositide 3-kinases-Akt (PI3K-Akt) survival cascade in colorectal cancer cells [52]. Δ9-THC (8 μM) and JWH-015 (8 μM) (synthetic CB2 agonist) diminished ascites’ development in an orthotopic model of hepatocellular carcinoma (HCC) and also reduced the growth of HepG2 and HuH-7-derived tumor xenografts. The two cannabinoids, through CB2 activation, subsequent Akt/mTORC inhibition, and AMPK activation, triggered autophagy stimulation that led to HCC apoptosis [53].

In a recent study about 10 compounds were selected from a synthetic cannabinoid library for their ability to reduce viability of several CRC cell lines characterized by different expression of mRNA levels of CB1, GPR55, and TRPV1 receptors. They showed that seven of the 10 selected compounds were selective for CRC cells but were unable to reduce the viability of HEK 293 or CCD 841 CoTr cells. Interestingly, treatment with Δ9-THC (10 μM) or CBD (10 μM) was either ineffective or much less potent and only partially efficacious. Moreover, treatment with CB1, CB2, GPR55, and/or TRPV1 antagonists (alone or in combination) failed to block the activity of the most potent identified compounds, suggesting that its action is independent of the activation of canonical receptors [54].

Our research group investigated the effect of rimonabant (SR141716), a CB1 receptor antagonist/inverse agonist, on colorectal carcinogenesis. Results showed that, starting from 2.5 μM, rimonabant inhibited CRC cell growth-inducing G2/M cell cycle arrest and mitotic catastrophe and decreased the number of ACF containing four or more crypts in AOM-treated mice [55]. We recently clarified that, in CRC, rimonabant exerts its anti-tumor action through Wnt/β-catenin canonical pathway inhibition, both in vitro and in vivo. We found that rimonabant (10 μM) acts on the β-catenin pathway, inhibiting transcriptional activity on T-cell factor/lymphoid enhancer factor (TCF/LEF) responsive elements and promoting its degradation and nuclear translocation. Noteworthy, we identified the β-catenin transcriptional co-activator, p300/KAT3B, as a direct target of rimonabant [56].

In subsequent work, we demonstrated that rimonabant-mediated inhibition of Wnt/β-catenin pathway impacts on chemoresistance and cancer stemness. In CRC, rimonabant strongly synergizes with 5-FU and, interestingly, in primary colon cancer stem cells it reduces CD133+/CD44+ population and spheroids’ formation. Of note, rimonabant did not show toxicity in 3D cultures of human healthy colon organoids [57]. Moreover, the combined synergic effect of rimonabant and oxaliplatin was able to block the proliferation of CRC cell lines [58]. The cannabinoid agonist WIN-55,212-2 (1–10 µM) inhibited the proliferation and induced apoptosis of both 5-FU-sensitive and -resistant human gastric cancer cells [59,60]. WIN-55,212-2 treatment was able to inhibit AKT activation, implicated in survival and migration, as well as downregulate Matrix metalloproteinases-2 (MMP-2) and VEGF-A expression, two extracellular factors involved in tumor invasiveness processes [59]. Taken together, this emerging evidence indicates that targeting the ECS could be an advantageous strategy to treat GI cancers.

## 4. Lung Cancer

Early studies evidenced the effects of *Cannabis* exposure on pulmonary functions and health. Although marijuana smoking might be responsible for lung epithelium hyperplasia and cellular disorganization [61], some cannabinoid compounds exhibited antitumorigenic properties. In the lung, both CB1 and CB2 receptors were expressed on structural cells and most leukocytes [62]. Human lung-resident macrophages constitutively express higher CB2 than CB1 at mRNA and protein levels, a pattern observed also in monocyte-derived macrophages despite the different functional activation of the receptors between tissue- and blood-derived macrophages [63]. Preet et al. first compared CB1/CB2 levels of non-small cell lung cancers (NSCLCs) to their normal counterparts, showing that lung carcinomas, like other malignancies, overexpress the receptors with CB1 found in the 24% (7 of 29) of cases and CB2 in the 55% (16 of 29) [64]. The first evidence of cannabinoids’ antiproliferative properties comes from Munson et al. who reported that both Δ9-THC, the major psychoactive *Cannabis* constituent, and cannabinol (CBN) retarded Lewis lung adenocarcinoma cell growth of primary cell culture and in the murine model after oral administration [65]. Among the cannabinoids, CBD has a low affinity for cannabinoid receptors and elicits its effects independently of them. In lung cancers, CBD acts up-regulating PPARγ levels directly and indirectly by increasing prostaglandin levels, which leads to a nuclear PPARγ accumulation and subsequent induction of apoptosis [5]. Despite the anandamide analog, Met-F-AEA did not show significant antitumorigenic effects when used alone in NSCLC in vitro and in vivo. Together with FAAH inhibitor URB597 it was effective in inhibiting epidermal growth factor receptor (EGFR) phosphorylation and its downstream signal transduction pathways. Met-F-AEA (10 μM) in combination with URB597 (0.2 μM) caused G0/G1 cell cycle arrest mediated apoptosis, which is shown by a reduction in the G1/S phase checkpoint markers cyclin D1 and cyclin-dependent kinase 4 (CDK4) and apoptotic markers caspase-9 and poly (ADP-ribose) polymerase (PARP) [66]. The Δ9-THC, at 10 μM, showed anti-metastatic activities in A549 and SW-1573, NSCLC cell lines expressing CB1 and CB2. The Δ9-THC was able to attenuate the EGF-induced morphological changes related to the migratory phenotype causing a decrease in cell motility and invasion in vitro. Furthermore, Δ9-THC inhibited in vivo tumor cell proliferation and vascularization. The observed effects were attributed to the reduction of signaling molecules, like FAK, ERK1/2, and Akt, involved in extracellular matrix (ECM) remodeling and cell survival [67]. CBD also revealed a potent anti-metastatic activity against lung cancer. Indeed, at very low concentrations (3 μM), CBD induced intracellular adhesion molecule 1 (ICAM-1) and TIMP1 levels, decreasing cellular migration [12] and increasing cancer cell lysis [68]. The mechanism has been associated with p38 and p42/44 MAPK phosphorylation as a direct consequence of CB1/CB2 activation [69]. In A549 cells, CBD treatment (1 μM) was also accompanied by the downregulation of the plasminogen activator inhibitor PAI-1, another important factor modulating lung cancer cell spreading. Therefore, PAI-1 overexpression or silencing in A549 led to a concentration-dependent up- and downregulation of invasiveness, respectively. The authors addressed a causal link between the CBD effects and PA1 secretion, demonstrating that treatment of A549 cells with recombinant PAI-1, at non-pro-invasive concentrations (0.01–0.1 ng/mL), reversed the anti-invasive effect of cannabidiol. The in vitro observations about the anti-metastatic activity of CBD was further confirmed in A549 xenografts that received 5 mg/kg cannabidiol intraperitoneally for 3 weeks [70]. In in vitro adenocarcinoma models, the FAAH inhibition mediated by URB597 enforced the Met-F-AEA effect in reducing migratory structures, like actin stress fibers and focal adhesions. Concomitantly, the URB597-Met-F-AEA combination reduced MMP2 and MMP9 secretion conferring invasion in xenograft tumors, thus confirming the in vitro findings [66]. More recently, Winkler et al. investigated the impact of two FAAH inhibitors (URB597, AA-5HT) and four FAAH substrates (AEA, 2-AG, OEA, PEA) on lung cancer cells spreading. FAAH inhibitors were shown to confer anti-invasive effects via the up-regulation of the matrix metalloproteinase inhibitor TIMP-1 and FAAH substrates mimicked the anti-invasive action of FAAH inhibitors, in agreement with previous evidence [17]. Similar results were obtained with the synthetic cannabinoids JWH-015 and WIN-55,212-2, CB2 and CB1/CB2 agonists, respectively, that from 0.1 to 2 μM significantly inhibited EGF- or serum-induced proliferation and were also able to confer rounded cellular shape, thus inhibiting migration and invasion of NSCLC cell lines and tumor growth and dissemination in murine models. The observed effects were reverted by CB1/CB2 antagonist, thus indicating a direct role of the endocannabinoid receptors in lung cancer progression [64]. Ravi et al. showed that JWH-015 may act on the tumor microenvironment influencing the crosstalk between the cancer cell and the host cell. In the epithelial cell line A549, JWH-015 reverted the mesenchymal character induced by EGF stimulation and, vice versa, in mesenchymal cell line CALU1 it up-regulates epithelial markers. Furthermore, JWH-015, through CB2 activation, blocked factors’ secretion by M2 tumor-associated macrophages co-cultured with lung adenocarcinoma cells A549 and inhibited their recruitment in vivo at the tumor site, thus attenuating the epithelial to mesenchymal transition [71]. Ramer et al. evaluated the impact of some cannabinoids on tumor-to-endothelial cell communication, playing a role in the angiogenic process. They observed that CBD, Δ9-THC, and Met-AEA or JWH-133, a CB2 agonist, decreased the migration and the sprout formation of HUVECs suspended in conditioned media of A549 lung cancer cells. Collectively, their data suggested that cannabinoids, through the activation of CB1 and CB2 receptors as well as TRPV1, increased the TIMP-1 release from lung cancer cells via activation and subsequent induction of intercellular adhesion molecule 1 (ICAM-1) expression, thereby altering the cancer cell microenvironment and suppressing the angiogenic potential of endothelial cells [70]. Moreover, the synthetic agonists, Arachidonyl-2′-chloroethylamide (ACEA) and JWH-133, were found to notably inhibit the release of angiogenic and lymphangiogenic factors, such as VEGF-A, VEGF-C, and angiopoietins, by human lung-resident macrophages and to modestly affect the secretion of the pro-inflammatory cytokine IL-6 [63].

## 5. Breast and Prostate Cancers

The effects of CBs may slow down tumor progression in breast cancer via G-protein coupled CB-receptors (CB-Rs), CB1-R and CB2-R. In breast cancer cell lines CB2 receptors are expressed at high levels with respect to the levels of CB1 receptors [72,73]. Moreover, CB2-R expression in breast cancer correlates with the tumor aggressiveness. Estrogen and/or progesterone receptor-negative tumors, more aggressive than tumors expressing steroid–hormone receptors, express higher levels of CB2-R and usually have a better prognosis [74]. Breast cancer cell lines, such as estrogen receptor ER-positive cell lines (MCF-7, ZR-75-1, and T47D), and ER-negative cell lines (MDA-MB-231, MDA-MB-468, and SK-BR3), are sensitive to the antiproliferative effects of CBD. This phytocannabinoid, at concentration ranging from 8.2 ± 0.3 μM to 10.6 ± 1.8 μM, inhibits the breast cancer cell proliferation through various mechanisms: (1) Blocks of cell cycle at the G1/S phase via CB1 and at the G2/M phase via CB2 activation, (2) induction of apoptosis by activation of the transcription factor jun-D, and (3) inhibition of AKT and increase of ROS generation [74]. It also induces autophagic death by increasing of endoplasmic reticulum stress, followed by the accumulation of microtubule-associated protein 1 light chain 3, (LC3-II) [75]. In HER2-overexpressing breast cancer cells, CBD arrested cancer cell proliferation in vitro and in vivo by inhibiting Akt and ERK signaling [74]. Furthermore, CBD restrict epidermal growth factor (EGF)-induced tumorigenic properties by inhibiting EGFR, Akt, ERK, and NF-κB signaling pathways as well as matrix metalloproteinase 2 and 9 in human breast cancer cells [76]. Additionally, CBD modulated the breast tumor microenvironment through a decrease of the cytokines production, as chemokine (C-C motif) ligand 3 (CCL3) and granulocyte-macrophage colony-stimulating factor (GM-CSF), which determined a reduction of the recruitment of total macrophages and an M2 macrophages polarization into the primary and secondary tumor sites, encouraging the tumor progression and metastasis to distant organs [76]. In advanced stages of breast cancer, CBD (1.5 μmol/L) reduced metastasis through down-regulation of the transcriptional regulator Id1, which plays a critical role in mediating breast cancer tumorigenicity [77]. In addition, CBD significantly increased activation of the transient receptor potential vanilloid type-2 (TRPV2), which allowed the uptake of doxorubicin (DOX) and apoptosis in triple-negative breast cancer cells (TNBC cells). Studies in vivo have shown that the combination of CBD and DOX significantly reduced the weight of TNBC tumors compared to those treated with CBD or DOX alone [78]. The metabolically stable analog of anandamide, Met-F-AEA (10 μM), was reported to inhibit the proliferation of the estrogen receptor-negative-MDA-MB-231 breast cancer cells, inducing an S phase cell cycle arrest correlated with DNA damage and Chk1 activation [79]. Anandamide inhibited also 3-hydroxy-3-methyl-glutaryl-coenzyme A reductase (HMG-CoA reductase) activity, thus affecting the pattern of expression of oncogenic prenylated proteins involved in the proliferation and metastatic potential of breast cancer cells, such as Ras and RhoA [80]. Indeed, anandamide reduced the invasiveness of highly metastatic MDA-MB-231 cells, inhibiting their migration through the RhoA signaling pathway, and inhibited cell migration via CB1 activation by affecting FAK/SRC/RhoA pathway [81]. The efficacy of 2-methyl-2′-F-anandamide (Met-F-AEA),has been maintained in the in vivo setting since it was able to reduce the number and dimension of metastatic nodes in a mouse model of metastatic spreading [81]. More, other synthetic cannabinoids, such as ACEA (at a concentration ranging from 50 nM to 200 nM) and AM251 (at a concentration ranging 10 to 40 nM) affected the invasive potential of breast cancer stem cells. Indeed, while ACEA, a selective CB1 agonist, decreased the invasive potential of breast cancer stem cells, AM251, a selective CB1 antagonist, promoted invasion, indicating that CB1 receptors are involved in the regulation of stem cell properties [82]. More, cannabinoids showed anti-angiogenic effects in breast cancer, decreasing or inhibiting the synthesis of pro-angiogenic factors such as VEGF. The Δ9-THC has shown to be harmful for tumor vascularization, reducing the number of blood vessels [83]. Lastly, Blasco-Benito et al. observed that HER2 interacts with CB2 receptors in breast cancer cells and the expression of these heteromers correlates with poor patient prognosis. The cannabinoid Δ9-THC disrupted HER2–CB2R complexes by selectively binding to CB2R, which led to the inactivation and degradation of HER2 through disruption of HER2–HER2 homodimers promoting antitumoral responses both in vitro and in vivo, which may constitute a new strategy to treat HER2+ breast tumors [84].

Natural and synthetic cannabinoids have been shown to inhibit cell growth in culture and experimental animal models of prostate cancer. Numerous observations have highlighted the ability of cannabinoids to inhibit prostate cancer cells’ viability/proliferation, as well as invasion and metastasis. Sarfaraz S. et al. showed that CB1 and CB2 are higher in human prostate cancer cells LNCaP and DU145 and PC3 cancer cells than in normal cells. Importantly, they also observed that WIN-55,212-2 treatment (CB1/CB2 agonist), at final concentrations of 1.0, 2.5, 5.0, 7.5, and 10.0 moL/L, inhibited cell growth of androgen-responsive LNCaP cells with a concomitant induction of apoptosis results in a dose- and time-dependent manner. In addition, WIN-55,212-2 treatment decreased protein and mRNA expression of androgen receptor and prostate-specific antigen (PSA) and protein expression of proliferating cell nuclear antigen (PCNA), and VEGF [85]. Anandamide (at 2 μM) induced a decrease of EGFR levels on LNCaP, DU145, and PC3 prostatic cancer cells via cannabinoid CB1 receptor subtype, causing an inhibition of the EGF-stimulated growth of these cells and apoptosis and/or necrosis [86]. Endogenous 2-AG (1 μmoL/L) inhibited the invasive ability of androgen-independent prostate cancer cells as PC3, DU-145, and LNCaP cells by a mechanism involving the CB1 receptor and through the inactivation of protein kinase A [87,88]. Morell et al. reported that the cannabinoid WIN-55,212-2 prevents neuroendocrine (NE) differentiation of LNCaP prostate cancer cells by inhibition of PI3K/Akt/mTOR activation and stimulation of AMPK [89]. Endocannabinoids, such as AEA, 2-AG, and methanandamide at final concentrations of 2.5, 5.0, and 10.0 μM, can impair the growth of prostate cancer cells through activation of apoptotic mechanisms, increase the levels of active caspase-3, and decrease the expression levels of Bcl-2. Furthermore, these effects are mediated by the modulation of the ERK and AKT signaling pathways [90]. CBD (at 0.5–7 µl/mL) inhibits the spheroid formation and dow-nregulates CB1 and CB2 receptors, VEGF, PSA, and pro-inflammatory cytokines IL-6/IL-8 in LNCaP prostate cancer stem cells [91]. In vivo, CBD-enriched cannabis extract (1–100 mg/kg^−1^) reduced tumor size in LNCaP-xenografted mice and enhanced the anticancer effect of bicalutamide (50 mg/kg^−1^), but not of docetaxel (5 mg/kg^−1^), while in DU-145 xenografts CBD was inactive alone but able to potentiate the effect of docetaxel [92]. Lastly, Chung et al. found that high CB1 receptors’ immunoreactivity is associated with a more severe form of the cancer at diagnosis and a poorer outcome [93].

## 6. Pancreatic and Thyroid Cancers

The antitumor properties of cannabinoids have been shown also in pancreatic cancers. Indeed, the presence of CB1 and CB2 receptors has been demonstrated in exocrine and endocrine pancreatic tissue and it is suggested that the ECS plays an important role in the regulation of pancreatic secretion [94,95,96,97,98]. In human islets of Langerhans, CB1 is densely located in glucagon-secreting alpha cells and less in insulin-secreting beta cells. CB2 is largely expressed in somatostatin-secreting delta cells but absent in alpha and beta cells [94]. In the rat, the expression of CB1 and CB2 has been demonstrated in pancreatic lobules (with a higher expression of CB1 compared to CB2) and pancreatic acini [98]. It was shown that CB1 and CB2 are overexpressed in human pancreatic tumor cell lines and biopsies and that cannabinoids selectively reduce the pancreatic cancer cell growth, both in vitro and in vivo, compared to pancreatic nontransformed cells [99].

Studies conducted on MiaPaCa2 and Panc1 human pancreatic cancer cell lines showed that Δ9-THC (2µM for MiaPaCa2 and 2.75 µM for Panc1) induced caspase-3 activation, characteristic of apoptotic cell death. In these pancreatic cancer cell models, Δ9-THC administration stimulated the de novo synthesis of ceramide that, in turn, led to the up-regulation of stress-regulated protein p8, increasing the cell apoptotic rate. The up-regulation of endoplasmic reticulum stress-related *atf-4* and *trb3* genes suggests their products as potential mediators of p8-dependent apoptotic effects. The Δ9-THC cytotoxic effects are prevented: (1) By blockade of the CB2 (but not of CB1) cannabinoid receptor, (2) pharmacologic inhibition of de novo ceramide synthesis, and (3) silencing of *p8* gene. These antitumor effects of CBs were confirmed also in tumor xenografts and orthotopic mice models. Moreover, in tumor orthotopic mice, the administration of the synthetic cannabinoid agonist WIN-55,212-2 (1.5 mg/Kg for 2 days, 2.25mg/Kg for 2 additional days, 3 mg/Kg for 10 additional days) reduced the growth and the spreading of pancreatic tumor cells [99].

It was also shown that the synthetic cannabinoids arachidonylcyclopropylamide (ACPA) (200µM) and GW405833 (GW) (200 µM), binding CB1 or CB2, respectively, induced ROS-mediated autophagy in Panc1 cell line. The oxidative stress in cannabinoid-treated Panc1 cells, increasing the AMP/ATP ratio, promoted the activation of AMPK, leading to the inhibition of energetic metabolism and autophagy. Indeed, after ACPA or GW treatment there was a general inhibition of glycolysis via decreasing of the key glycolytic enzymes, glyceraldehyde-3-phosphate dehydrogenase (GAPDH) and pyruvate kinase isozymes M2 (PKM2). GAPDH has been identified as a key redox-sensitive protein, and its activity is largely affected by covalent oxidative modifications at its highly reactive Cys152. These modifications stimulate the nuclear translocation of GAPDH, often leading to autophagy activation. Furthermore, AMPK has been shown to stimulate the GAPDH translocation into the nuclei [100]. It was also demonstrated that the combination of the standard chemotherapeutic agent gemcitabine (GEM) and GW or ACPA increases the pancreatic tumor autophagic cell death, induced by ROS production [101]. The endocannabinoid 2-AG exerts direct antitumor effects via inhibiting pancreatic cancer cell proliferation both in vitro and pancreatic ductal adenocarcinoma orthotopic animal models (daily intraperitoneal injection of 2-AG 20 mg/kg). In addition, in vivo studies shown that 2-AG induces immunomodulatory effects in PC environment, leading to the dendritic cell maturation and promoting an immunosuppressive microenvironment via increasing of myeloid-derived suppressor cells. Inhibition of tumor proliferation, as well as immunomodulatory effects of 2-AG, were prevented by CB1 receptor antagonists but not by CB2 receptor antagonists, suggesting the involvement of CB1-mediated mechanisms [102]. The effect of the CBs mediated by CB receptors is not the only mechanism involved in the inhibition of pancreatic tumor cell growth; indeed, studies reported that AM251 and other CBs induce cytotoxic effects via a receptor-independent mechanism in Mia PaCa2 [103].

The inhibitory effects of CBs were also demonstrated in thyroid cancer (TC). Enhanced CB1 and CB2 receptor expression were correlated with malignant thyroid lesions. In particular, high CB2 expression levels were significantly correlated with higher malignancy of TC and presence of metastases in lymph node, and they would seem associated with increased risk of cancer recurrence. The altered expression of CB receptors may be involved in thyroid malignant transformation and progression and could serve as prognostic factor. In this context, CBs receptors, especially CB2, may represent a potential therapeutic target to suppress TC progression [104].

It was reported that Met-F-AEA (10 µM), a metabolically stable analog of anandamide, interacting with the receptor CB1, is able to inhibit TC cell growth and increase the apoptotic rate via activation of p53 signaling and expression of p21^waf1^ [105].

Moreover, CB2 activation induced apoptosis in anaplastic TC cell lines. The administration of CB2 agonist JWH133 (daily intratumoral injection of 50 μg/mL for 3 weeks) led to a considerable regression of thyroid tumors generated in nude mice by inoculation of the TC cells ARO/CB2 [106]. Endocannabinoids were effective against cancer cells with activated BRAF/ERK and/or TrkA signaling, suggesting their potential utility for the treatment of BRAF-positive papillary thyroid carcinoma (PTC) and TrkA-positive medullary thyroid cancer [107].

## 7. Brain Cancer

Glioblastoma multiforme (GBM) is the most aggressive form and constitutes 15.6% of all primary brain tumors. Treatment options remain very limited due to their aggressiveness and heterogeneity. Despite multimodal therapy consisting of surgery, radiation, and chemotherapy, only 28.4% of patients survive one year and 3.4% survive to five years. This highlights the need for new therapeutic strategies. In the last few years, CBs, and in particular Δ9-THC and CBD, have exhibited anticancer activity in preclinical models of cancer and specifically in glioma [108]. The CB1 receptor is expressed mainly in the brain at very high levels in the basal ganglia, hippocampus, cerebellum, and cortex. The CB2 receptor is expressed mainly in peripheral immune cells. However, strong evidence shows that CB2 receptors are moderately expressed and function in specific brain areas [109]. CB1 and CB2 receptors are also expressed in GBM tumors. These have been detected in GBM cell lines, primary cells from tumors, and biopsies of GBM. CB2 expression positively correlates with malignancy grade. It has been reported that CB1 expression is unchanged, decreased, or even increased in GBM compared to control tissues [110]. In orthotopic and subcutaneous animal models of glioma, the treatment with cannabinoids resulted in a significant reduction of tumor growth [111]. Upon cannabinoid treatment, there was an increase in the activation of apoptotic cell death through the consequent activation of different pathways. In fact, cannabinoids phosphorilate BAD proapoptotic protein with a consequent loss of integrity of the outer mitochondrial membrane. In addition, cannabinoids activated the intrinsic apoptosis pathway after an increase of ceramide which, in turn, inhibited the PI3K/Akt and Raf1/MEK/ERK pro-survival pathways, thereby allowing BAD to translocate to the mitochondria. CBs were also shown to trigger apoptosis via ceramide-mediated cell death and via oxidative stress [112]. Specifically, in glioma cells, CBD led to a production of reactive oxygen species (ROS), glutathione (GSH) depletion, and caspase-9, -8, and -3 activation. Furthermore, it was observed a significant increase in the formation of ROS after the combined treatment of GBM cells with Δ9-THC and CBD, which was also linked to a later induction of apoptosis [113]. Recently, however, Scott et al. showed that, while CBD treatment of glioma cells induces a significant increase in ROS production, this phenomenon is accompanied by an upregulation of a large number of genes belonging to the heat-shock protein (HSP) super-family with consequent decrease of the cytotoxic effect of CBD. For this reason, the possible inclusion of HSP inhibitors might enhance the antitumor effects of cannabinoids in glioma/GBM treatment [114]. Apart from a direct killing effect on tumor cells, cannabinoids also work in the direction of inhibiting tumor cell proliferation. Marcu et al. showed that treatment of GBM cells with Δ9-THC (0.1 µM) and/or CBD (0.1 µM) increased the population of cells in the G0/G1 phase and G2/M phase while decreasing the number of cells in the S phase [113]. Galanti et al. were able to characterize some of the molecular mechanisms involved in cannabinoid-induced cell cycle arrest in G0-G1 phase and found that Δ9-THC (0–50 µg/mL) decreased the levels of E2 Transcription Factor 1 (E2F1) and cyclin A (two proteins that promote cell cycle progression) while upregulating the levels of the cell cycle inhibitor p16INK4A [115]. A tumor-specific GBM cytostatic/cytotoxic effect of cannabinoids is not the only aspect to investigate recently. Several studies showed that cannabinoids were also able to inhibit tumor angiogenesis. For instance, Blázquez et al. [116] found that local administration of a nonpsychoactive cannabinoid JWH133 (50 μg/day) to mice inhibited angiogenesis of malignant gliomas. Moreover, they were able also to demonstrate that local administration of Δ9-THC reduced pro-angiogenic VEGF levels in two patients with recurrent GBM [117]. Solinas et al. demonstrated that CBD induced endothelial cell cytostasis in vitro and inhibited endothelial cell migration and angiogenesis in vivo. They have shown that these effects were accompanied by a downregulation of pro-angiogenic factors MMP2, MMP9, platelet-derived growth factor-AA (PDGF-AA), urokinase-type plasminogen activator (uPA), endothelin-1 (ET-1), and chemokine (C-X-C motif) ligand 16 (CXCL16) [118]. Most studies found that the agonistic stimulation via CB receptors is responsible for the antitumor effects of cannabinoids, suggesting that CB1 agonists might also be useful in glioma therapy. Specifically, Ciaglia et al. [119] found that the pharmacological inactivation of CB1 by SR141716 (20 μM) led to the inhibition of glioma cell growth through cell proliferation arrest and induction of caspase-dependent apoptosis. Additionally, SR141716 upregulated the expression of natural killer group 2D (NKG2D) ligands (MHC class I chain-related protein A MICA and MHC class I chain-related protein B MICB) on the surface of glioma cells via signal transducer and activator of transcription 3 (STAT3) inactivation leading to a consequent increase of MICA/B levels and enhancing the recognition of glioma cells by NK-cells. Notably, SR141716-induced MICA/B upregulation directly correlated with the degree of CB1 expression and occurred only in malignant glioma cells but not in normal human astrocytes [119]. Taken together, these findings suggest that CB1 specific agonists, at least for certain subsets of GBM with high expression of CB1, might be useful in multimodal therapeutic strategies. Glioma cells are very adept at infiltrating the surrounding healthy brain tissue and spreading through the brain parenchyma [120]. The role of cannabinoids in GBM migration and invasion is not still well characterized. For instance, Soroceanu et al. [121] have observed that CBD inhibits the invasion of GBM cells through organotypic brain slices. This anti-invasive effect was attributed to the inhibition of Id-1 (inhibitor of differentiation/DNA binding) that is a member of the helix-loop-helix protein family expressed in actively proliferating cells. The expression of Id1 was decreased by CBD treatment, as observed in several GBM cell lines, in ex-vivo primary GBM cells and orthotopic xenograft murine models [121]. Solinas et al. found that CBD (1 μM) significantly inhibited GBM invasion even at low concentrations, which were otherwise not sufficient to induce tumor cell death. The authors further demonstrated that CBD treatment of GBM cells significantly downregulated MMPs and TIMPs (in particular MMP-9 and TIMP-4), the major proteins associated with tumor invasion [122]. Furthermore, glioma Δ9-THC treatment can downregulate TIMP-1 and MMP-2, showing that these effects were mediated via ceramide accumulation and activation of p8 stress protein and, interestingly, it was observed in glioma-bearing mice as well as in two patients with recurrent GBM who had received intra-tumor injections with Δ9-THC [123]. The high recurrence rates of GBM tumors are partly related to the presence of glioma stem-like cells (GSCs) and a major challenge for GBM treatment is the resistance to therapy of the recurrent tumors. This phenomenon is under control of a subpopulation of GSCs, through multiple mechanisms, such as alteration of DNA damage response, hypoxic microenvironment, notch signaling pathway, or multidrug resistance [124]. GSCs express cannabinoid receptors, CB1 and CB2, as well as other components of the endocannabinoid system. Data from a gene array show that cannabinoid agonists HU-210 and JWH133 (30 nM both) altered the expression of genes involved in stem cell proliferation and differentiation. Indeed, in cannabinoid-treated GSCs an increase of S-100ß and glial fibrillary acidic protein GFAP expression was detected and at the same time the downregulation of the neuroepithelial progenitor marker nestin. Furthermore, cannabinoid treatment decreased neurosphere formation and cell proliferation in secondary xenografts mice models [125]. The differentiation of GSCs has been recently connected to the expression levels of the transcription factor Aml-1a. The upregulation of Aml-1a has been found during GSCs’ differentiation while Aml-1a knock-down was able to restore a stem cell phenotype in differentiated GSCs. Interestingly, treatment of GSCs with CBD (10 µM) upregulated the expression of Aml-1a in a TRPV2- and PI3K/Akt-dependent manner, thereby inducing autophagy and abrogating the chemoresistance of GSCs to carmustine (BCNU, bis chlorethyl-nitroso-urea) therapy [126]. CBD was shown to inhibit the self-renewal of GSCs via activation of the p38-MAPK pathway and downregulation of key stem cell mediators such as Sox2, Id1, and p-STAT3 [127]. Moreover, currently, a Phase 2 placebo-controlled clinical study (a clinical trial NCT01812603), based on treatment of Δ9-THC: CBD (12 sprays per day delivering 100 μL of a solution containing 27 mg/mL Δ9-THC and 25 mg/mL CBD) in combination with dose-intense temozolomide (TMZ) in 21 patients with recurrent GBM showed a median survival over 662 days compared with 369 days in the control group [128,129]. In conclusion, the high resistance of GBM to standard therapy consisting of surgical resection and radiotherapy in addition to adjuvant chemotherapy and TMZ are not sufficient anymore to get an opportune therapy [130]. For this reason a detailed understanding of cannabinoid-induced molecular mechanisms and pharmacological effects is required. Moreover, cancers affecting the central nervous system (CNS) should be regarded as a major health challenge due to the current lack of effective treatments given the hindrance to brain drug delivery imposed by the blood–brain barrier since the BBB truly hinders the distribution to the CNS of most drug substances administered systemically. In consequence, high doses of chemotherapy are often required to achieve therapeutically meaningful levels in the CNS and this causes severe toxicity to peripheral tissues. Therefore, there is a need for developing effective strategies of brain drug delivery that overcome biodistribution and pharmacokinetic limitations that account for treatment failure. In this regard, we hypothesized that cannabinoids hold great promise for brain active targeting. The high BBB transcytosis efficacy of cannabinoids that can be used also for delivery carriers arises as an alternative to enhance the passage across the BBB. In particular, Torres-Suárez Ana I. et al. decorated lipid nanocapsules (LNCs) with CBD carriers against glioma cells, enhancing the passage of LNCs across the blood–brain barrier [131]. All these results of clinical investigations show the importance of cannabinoid translational research. The cannabinoids can enhance chemotherapeutic agents’ activity, showing a lot of anti-neoplastic activities in GBM: Attenuating resistance to programmed cell death, neoangiogenesis, tissue invasion, or stem cell-induced replicative immortality.

The effects of all mentioned cannabinoids are summarized in Table S1 in supplementary materials.

## 8. Conclusions

The literature strongly suggests a role for the ECS in the pathogenesis of cancer. It is evident that cannabinoids target key signaling pathways affecting all the hallmarks of cancer. However, they complement the conventional chemotherapeutic regimens currently used preventing pain, nausea, and vomiting. Further studies will be necessary to fully elucidate their clinical relevance for cancer treatment. More intensive basic research will allow us to better understand the intracellular signaling pathways in cannabinoid anticancer action, identify intracellular factors modulated by cannabinoids, and discern tumors sensitive or resistant to cannabinoids. Results from these studies are essential to clarify whether cannabinoids could be helpful in cancer treatment. An interesting idea is their synergistic interaction with some conventional cytostatic drugs as well as their capacity to suppress metastasis and angiogenesis. Indeed, several studies described that Δ9-THC and CBD increased the cytostatic effects of chemotherapeutic drugs, such as the combination of CBD with DOX in vivo mouse model of triple-negative breast cancer (TNBC) that showed significantly higher activity than DOX alone and no obvious signs of toxicity were observed in mice treated with combination treatment. More, the promising data from studies on animal models of glioblastoma treated with Δ9-THC and temozolomide have led to clinical trials using combinatorial treatments of nabiximols and temozolomide in patients with recurrent glioblastoma. Taken together, cannabinoids and compounds modulating the endocannabinoid system may enrich the range of used chemotherapeutic agents as a pharmacotherapeutic option for cancer treatment. In the coming years, the discoveries on the endocannabinoid system may allow the development of more efficacious and safer compounds. Moreover, observations obtained from next-generation sequencing of tumors can best identify potent combinations of cannabinoids formulations and tumors with specific characteristics. These new approaches could lead to the identification of cannabinoid therapy-associated biomarkers in tumor biopsies or, ideally, high levels of resistance factors released by cancer cells. These biomarkers would conceivably relate to the expression and activity of cannabinoid receptors and then define the sensitivity of a particular tumor to cannabinoid-based therapies. Future studies should also emphasize investigations of administration routes, delivery schedules, and absorption of medicinal cannabis to further explore its application in cancer management, allowing a better assessment of the efficacy of cannabinoids in the fight of cancer.

## Supplementary Materials

Supplementary materials can be found at https://www.mdpi.com/1422-0067/21/3/747/s1.

## References

[1] Andre C.M., Hausman J.F., Guerriero G. (2016). Cannabis sativa: The plant of the thousand and one molecules. Front. Plant Sci..

[2] Thomas A., Baillie G.L., Phillips A.M., Razdan R.K., Ross R.A., Pertwee R.G. (2007). Cannabidiol displays unexpectedly high potency as an antagonist of CB1 and CB2 receptor agonists in vitro. B. J. Pharmacol..

[3] Khan M.I., Sobocinska A.A., Czarnecka A.M., Krol M., Botta B., Szczylik C. (2016). The therapeutic aspects of the endocannabinoid system (ECS) for cancer and their development: From nature to laboratory. Curr. Pharmaceut. Des..

[4] Maurya N., Velmurugan B.K. (2018). Therapeutic applications of cannabinoids. Chem. Biol. Interact..

[5] Ramer R., Schwarz R., Hinz B. (2019). Modulation of the Endocannabinoid System as a Potential Anticancer Strategy. Front. Pharmacol..

[6] Muller C., Morales P., Reggio P.H. (2019). Cannabinoid Ligands Targeting TRP Channels. Front. Mol. Neurosci..

[7] Javid F.A., Phillips R.M., Afshinjavid S., Verde R., Ligresti A. (2016). Cannabinoid pharmacology in cancer research: A new hope for cancer patients?. Eur. J. Pharmacol..

[8] Hinz B., Ramer R. (2019). Anti-tumour actions of cannabinoids. Br. J. Pharmacol..

[9] Fonseca B.M., Teixeira N.A., Correia-da-Silva G. (2017). Cannabinoids as Modulators of Cell Death: Clinical Applications and Future Directions. Rev. Physiol. Biochem. Pharmacol..

[10] Moreno E., Cavic M., Krivokuca A., Casadó V., Canela E. (2019). The Endocannabinoid System as a Target in Cancer Diseases: Are We There Yet?. Front. Pharmacol..

[11] Laezza C., D’Alessandro A., Paladino S., Malfitano M.A., Proto M.C., Gazzerro P., Pisanti S., Santoro A., Ciaglia E., Bifulco M. (2012). Anandamide inhibits the Wnt/β-catenin signaling pathway in human breast cancer MDA MB 231 cells. Eur. J. Cancer..

[12] Velasco G., Sánchez C., Guzmán M. (2016). Anticancer mechanisms of cannabinoids. Curr. Oncol..

[13] Holland M.L., Lau D.T., Allen J.D., Arnold J.C. (2007). The multidrug transporter ABCG2 (BCRP) is inhibited by plant-derived cannabinoids. Br. J. Pharmacol..

[14] Vago R., Bettiga A., Salonia A., Ciuffreda P., Ottria R. (2017). Development of new inhibitors for N-acylethanolamine-hydrolyzing acid amidase as promising tool against bladder cancer. Bioorg. Med. Chem..

[15] Hamtiaux L., Hansoulle L., Dauguet N., Muccioli G.G., Gallez B., Lambert D.M. (2011). Increasing antiproliferative properties of endocannabinoids in N1E-115 neuroblastoma cells through inhibition of their metabolism. PLoS ONE.

[16] Izzo A.A., Aviello G., Petrosino S., Orlando P., Marsicano G., Lutz B., Borrelli F., Capasso R., Nigam S., Capasso F. (2008). Increased endocannabinoid levels reduce the development of precancerous lesions in the mouse colon. J. Mol. Med..

[17] Winkler K., Ramer R., Dithmer S., Ivanov I., Merkord J., Hinz B. (2016). Fatty acid amide hydrolase inhibitors confer anti-invasive and antimetastatic effects on lung cancer cells. Oncotarget.

[18] Ma M., Bai J., Ling Y., Chang W., Xie G., Li R., Wang G., Tao K. (2016). Monoacylglycerol lipase inhibitor JZL184 regulates apoptosis and migration of colorectal cancer cells. Mol. Med. Rep..

[19] Pisanti S., Picardi P., D’Alessandro A., Laezza C., Bifulco M. (2013). The endocannabinoid signaling system in cancer. Trends Pharmacol. Sci..

[20] Pagano E., Borrelli F., Orlando P., Romano B., Monti M., Morbidelli L., Aviello G., Imperatore R., Capasso R., Piscitelli F. (2017). Pharmacological inhibition of MAGL attenuates experimental colon carcinogenesis. Pharmacol. Res..

[21] Rodrigues T., Sieglitz F., Bernardes G.J. (2016). Natural product modulators of transient receptor potential (TRP) channels as potential anti-cancer agents. Chem. Soc. Rev..

[22] O’Sullivan S.E. (2016). An update on PPAR activation by cannabinoids. Br. J. Pharmacol..

[23] Ramer R., Heinemann K., Merkord J., Rohde H., Salamon A., Linnebacher M., Hinz B. (2013). COX-2 and PPAR-γ confer cannabidiol-induced apoptosis of human lung cancer cells. Mol. Cancer Ther..

[24] Morales P., Reggio P.H. (2017). An Update on Non-CB1, Non-CB2 Cannabinoid Related G-Protein-Coupled Receptors. Cannabis Cannabinoid. Res..

[25] Piñeiro R., Maffucci T., Falasca M. (2011). The putative cannabinoid receptor GPR55 defines a novel autocrine loop in cancer cell proliferation. Oncogene.

[26] Falasca M., Ferro R. (2016). Role of the lysophosphatidylinositol/GPR55 axis in cancer. Adv. Biol Regul..

[27] Moreno E., Andradas C., Medrano M., Caffarel M.M., Pérez-Gómez E., Blasco-Benito S., Gómez-Cañas M., Pazos M.R., Irving A.J., Lluís C. (2014). Targeting CB2-GPR55 receptor heteromers modulates cancer cell signaling. J. Biol. Chem..

[28] Coke C.J., Scarlett K.A., Chetram M.A., Jones K.J., Sandifer B.J., Davis A.S., Marcus A.I., Hinton C.V. (2016). Simultaneous Activation of Induced Heterodimerization between CXCR4 Chemokine Receptor and Cannabinoid Receptor 2 (CB2) Reveals a Mechanism for Regulation of Tumor Progression. J. Biol. Chem..

[29] Scarlett K.A., White E.Z., Coke C.J., Carter J.R., Bryant L.K., Hinton C.V. (2018). Agonist-induced CXCR4 and CB2 Heterodimerization Inhibits Gα13/RhoA-mediated Migration. Mol. Cancer Res..

[30] Pérez-Gómez E., Andradas C., Blasco-Benito S., Caffarel M.M., García-Taboada E., Villa-Morales M., Moreno E., Hamann S., Martín-Villar E., Flores J.M. (2015). Role of cannabinoid receptor CB2 in HER2 pro-oncogenic signaling in breast cancer. J. Natl. Cancer Inst..

[31] Pesce M., D’Alessandro A., Borrelli O., Gigli S., Seguella L., Cuomo R., Esposito G., Sarnelli G. (2018). Endocannabinoid-related compounds in gastrointestinal diseases. J. Cell Mol. Med..

[32] Gustafsson S.B., Palmqvist R., Henriksson M.L., Dahlin A.M., Edin S., Jacobsson S.O., Öberg Å., Fowler C.J. (2011). High tumour cannabinoid CB1 receptor immunoreactivity negatively impacts disease-specific survival in stage II microsatellite stable colorectal cancer. PLoS ONE.

[33] Chen L., Chen H., Li Y., Li L., Qiu Y., Ren J. (2015). Endocannabinoid and ceramide levels are altered in patients with colorectal cancer. Oncol Rep..

[34] Martinez-Martinez E., Gomez I., Martin P., Sánchez A., Román L., Tejerina E., Bonilla F., Merino A.G., de Herreros A.G., Provencio M. (2015). Cannabinoids receptor type 2, CB2, expression correlates with human colon cancer progression and predicts patient survival. Oncoscience.

[35] Cianchi F., Papucci L., Schiavone N., Lulli M., Magnelli L., Vinci M.C., Messerini L., Manera C., Ronconi E., Romagnani P. (2008). Cannabinoid receptor activation induces apoptosis through tumor necrosis factor alpha-mediated ceramide de novo synthesis in colon cancer cells. Clin. Cancer Res..

[36] Hasenoehrl C., Feuersinger D., Sturm E.M., Bärnthaler T., Heitzer E., Graf R., Grill M., Pichler M., Beck S., Butcher L. (2018). G protein-coupled receptor GPR55 promotes colorectal cancer and has opposing effects to cannabinoid receptor 1. Int. J. Cancer.

[37] Wang D., Wang H., Ning W., Backlund M.G., Dey S.K., DuBois R.N. (2008). Loss of cannabinoid receptor 1 accelerates intestinal tumor growth. Cancer Res..

[38] Jung C.K., Kang W.K., Park J.M., Ahn H.J., Kim S.W., Taek O.S., Choi K.Y. (2013). Expression of the cannabinoid type I receptor and prognosis following surgery in colorectal cancer. Oncol. Lett..

[39] Suk K.T., Mederacke I., Gwak G.Y., Cho S.W., Adeyemi A., Friedman R., Schwabe R.F. (2016). Opposite roles of cannabinoid receptors 1 and 2 in hepatocarcinogenesis. Gut.

[40] Tutino V., Caruso M.G., De Nunzio V., Lorusso D., Veronese N., Gigante I., Notarnicola M., Giannelli G. (2019). Down-Regulation of Cannabinoid Type 1 (CB1) Receptor and its Downstream Signaling Pathways in Metastatic Colorectal Cancer. Cancers.

[41] Fraguas-Sánchez A.I., Martín-Sabroso C., Torres-Suárez A.I. (2018). Insights into the effects of the endocannabinoid system in cancer: A review. Br. J. Pharmacol..

[42] Ortega A., García-Hernández V.M., Ruiz-García E., Meneses-García A., Herrera-Gómez A., Aguilar-Ponce J.L., Montes-Servín E., Prospero-García O., Del Angel S.A. (2016). Comparing the effects of endogenous and synthetic cannabinoid receptor agonists on survival of gastric cancer cells. Life Sci..

[43] DeMorrow S., Francis H., Gaudio E., Venter J., Franchitto A., Kopriva S., Onori P., Mancinelli R., Frampton G., Coufal M. (2008). The endocannabinoid anandamide inhibits cholangiocarcinoma growth via activation of the non canonicalWnt signaling pathway. Am. J. Physio. Gastrointest. Liver Physiol..

[44] Huang L., Ramirez J.C., Frampton G.A., Golden L.E., Quinn M.A., Pae H.Y., Horvat D., Liang L., DeMorrow S. (2011). Anandamide exerts its antiproliferative actions on cholangiocarcinoma by activation of the GPR55 receptor. Lab. Invest..

[45] Proto M.C., Gazzerro P., Di Croce L., Santoro A., Malfitano A.M., Pisanti S., Laezza C., Bifulco M. (2012). Interaction of endocannabinoid system and steroid hormones in the control of colon cancer cell growth. J. Cell Physiol..

[46] Fiore D., Proto M.C., Pisanti S., Picardi P., Pagano Zottola A.C., Butini S., Gemma S., Casagni A., Laezza C., Vitale M. (2016). Antitumor effect of pyrrolo-1,5-benzoxazepine-15 and its synergistic effect with Oxaliplatin and 5-FU in colorectal cancer cells. Cancer Biol. Ther..

[47] Zhang X., Qin Y., Pan Z., Li M., Liu X., Chen X., Qu G., Zhou L., Xu M., Zheng Q. (2019). Cannabidiol Induces Cell Cycle Arrest and Cell Apoptosis in Human Gastric Cancer SGC-7901 Cells. Biomolecules.

[48] Kargl J., Andersen L., Hasenöhrl C., Feuersinger D., Stančić A., Fauland A., Magnes C., El-Heliebi A., Lax S., Uranitsch S. (2016). GPR55 promotes migration and adhesion of colon cancer cells indicating a role in metastasis. Br. J. Pharmacol..

[49] Aviello G., Romano B., Borrelli F., Capasso R., Gallo L., Piscitelli F., Di Marzo V., Izzo A.A. (2012). Chemopreventive effect of the non-psychotropic phytocannabinoid cannabidiol on experimental colon cancer. J. Mol. Med..

[50] Jeong S., Yun H.K., Jeong Y.A., Jo M.J., Kang S.H., Kim J.L., Kim D.Y., Park S.H., Kim B.R., Na Y.J. (2019). Cannabidiol-induced apoptosis is mediated by activation of Noxa in human colorectal cancer cells. Cancer Lett..

[51] Honarmand M., Namazi F., Mohammadi A., Nazifi S. (2019). Can cannabidiol inhibit angiogenesis in colon cancer?. Comp. Clin. Path..

[52] Greenhough A., Patsos H.A., Williams A.C., Paraskeva C. (2007). The cannabinoid delta (9)-tetrahydrocannabinol inhibits RAS-MAPK and PI3K-AKT survival signaling and induces BAD-mediated apoptosis in colorectal cancer cells. Int. J. Cancer.

[53] Vara D., Morell C., Rodríguez-Henche N., Diaz-Laviada I. (2013). Involvement of PPARgamma in the antitumoral action of cannabinoids on hepatocellular carcinoma. Cell Death. Dis..

[54] Raup-Konsavage W.M., Johnson M., Legare C.A., Yochum G.S., Morgan D.J., Vrana K.E. (2018). Synthetic cannabinoid activity against colorectal cancer cells. Cannabis. Cannabinoid. Res..

[55] Santoro A., Pisanti S., Grimaldi C., Izzo A.A., Borrelli F., Proto M.C., Malfitano A.M., Gazzerro P., Laezza C., Bifulco M. (2009). Rimonabant inhibits human colon cancer cell growth and reduces the formation of precancerous lesions in the mouse colon. Int. J. Cancer.

[56] Proto M.C., Fiore D., Piscopo C., Franceschelli S., Bizzarro V., Laezza C., Lauro G., Feoli A., Tosco A., Bifulco G. (2017). Inhibition of Wnt/β-Catenin pathway and Histone acetyltransferase activity by Rimonabant: A therapeutic target for colon cancer. Sci. Rep..

[57] Fiore D., Ramesh P., Proto M.C., Piscopo C., Franceschelli S., Anzelmo S., Medema J.P., Bifulco M., Gazzerro P. (2018). Rimonabant Kills Colon Cancer Stem Cells without Inducing Toxicity in Normal Colon Organoids. Front. Pharmacol..

[58] Gazzerro P., Malfitano A.M., Proto M.C., Santoro A., Pisanti S., Caruso M.G., Notarnicola M., Messa C., Laezza C., Misso G. (2010). Synergistic inhibition of human colon cancer cell growth by the cannabinoid CB1 receptor antagonist rimonabant and oxaliplatin. Oncol. Rep..

[59] Xian X.S., Park H., Cho Y.K., Lee I.S., Kim S.W., Choi M.G., Chung I.S., Han K.H., Park J.M. (2010). Effect of a synthetic cannabinoid agonist on the proliferation and invasion of gastric cancer cells. J. Cell Biochem..

[60] Xian X.S., Park H., Choi M.G., Park J.M. (2013). Cannabinoid receptor agonist as an alternative drug in 5-fluorouracil-resistant gastric cancer cells. Anticancer Res..

[61] Tashkin D.P., Roth M.D. (2019). Pulmonary effects of inhaled cannabis smoke. Am. J. Drug Alcohol Abuse.

[62] Turcotte C., Blanchet M.R., Laviolette M., Flamand N. (2016). Impact of Cannabis, Cannabinoids, and Endocannabinoids in the Lungs. Front. Pharmacol..

[63] Staiano R.I., Loffredo S., Borriello F., Iannotti F.A., Piscitelli F., Orlando P., Secondo A., Granata F., Lepore M.T., Fiorelli A. (2016). Human lung-resident macrophages express CB1 and CB2 receptors whose activation inhibits the release of angiogenic and lymphangiogenic factors. J. Leukoc. Biol..

[64] Preet A., Qamri Z., Nasser M.W., Prasad A., Shilo K., Zou X., Groopman J.E., Ganju R.K. (2011). Cannabinoid receptors, CB1 and CB2, as novel targets for inhibition of non-small cell lung cancer growth and metastasis. Cancer Prev. Res..

[65] Munson A.E., Harris L.S., Friedman M.A., Dewey W.L., Carchman R.A. (1975). Antineoplastic activity of cannabinoids. J. Natl. Cancer Inst..

[66] Ravi J., Sneh A., Shilo K., Nasser M.W., Ganju R.K. (2014). FAAH inhibition enhances anandamide mediated anti-tumorigenic effects in non-small cell lung cancer by downregulating the EGF/EGFR pathway. Oncotarget.

[67] Preet A., Ganju R.K., Groopman J.E. (2008). Delta9-Tetrahydrocannabinol inhibits epithelial growth factor-induced lung cancer cell migration in vitro as well as its growth and metastasis in vivo. Oncogene.

[68] Haustein M., Ramer R., Linnebacher M., Manda K., Hinz B. (2014). Cannabinoids increase lung cancer cell lysis by lymphokine-activated killer cells via upregulation of ICAM-1. BiochemPharmacol.

[69] Pisanti S., Malfitano A.M., Ciaglia E., Lamberti A., Ranieri R., Cuomo G., Abate M., Faggiana G., Proto M.C., Fiore D. (2017). Cannabidiol: State of the art and new challenges for therapeutic applications. Pharmacol. Ther..

[70] Ramer R., Rohde A., Merkord J., Rohde H., Hinz B. (2010). Decrease of plasminogen activator inhibitor-1 may contribute to the anti-invasive action of cannabidiol on human lung cancer cells. Pharm. Res..

[71] Ravi J., Elbaz M., Wani N.A., Nasser M.W., Ganju R.K. (2016). Cannabinoid receptor-2 agonist inhibits macrophage induced EMT in non-small cell lung cancer by downregulation of EGFR pathway. Mol. Carcinog..

[72] Caffarel M.M., Sarrió D., Palacios J., Guzmán M., Sánchez C. (2006). ∆9-Tetrahydrocannabinol Inhibits Cell Cycle Progression in Human Breast Cancer Cells through Cdc2 Regulation. Cancer Res..

[73] McKallip R.J., Nagarkatti M., Nagarkatti P.S. (2005). Delta-9-tetrahydrocannabinol enhances breast cancer growth and metastasis by suppression of the antitumor immune response. J. Immunol..

[74] Kisková T., Mungenast F., Suváková M., Jäger W., Thalhammer T. (2019). Future Aspects for Cannabinoids in Breast Cancer Therapy. Int. J. Mol. Sci..

[75] Shrivastava A., Kuzontkoski P.M., Groopman J.E., Prasad A. (2011). Cannabidiol induces programmed cell death in breast cancer cells by coordinating the cross-talk between apoptosis and autophagy. Mol. Cancer.

[76] Elbaz M., Nasser MW., Ravi J., Wani NA., Ahirwar DK., Zhao H., Oghumu S., Satoskar A.R., Shilo K., Carson W.E. (2015). Modulation of the tumor microenvironment and inhibition of EGF/EGFR pathway: Novel anti-tumor mechanisms of Cannabidiol in breast cancer. Mol. Oncol..

[77] McAllister S.D., Christian R.T., Horowitz M.P., Garcia A., Desprez P.Y. (2007). Cannabidiol as a novel inhibitor of Id-1 gene expression in aggressive breast cancer cells. Mol. Cancer.

[78] Elbaz M., Ahirwar D., Xiaoli Z., Zhou X., Lustberg M., Nasser M.W., Shilo K., Ganju R.K. (2016). TRPV2 is a novel biomarker and therapeutic target in triple negative breast cancer. Oncotarget.

[79] Laezza C., Pisanti S., Crescenzi E., Bifulco M. (2006). Anandamide inhibits Cdk2 and activates Chk1 leading to cell cycle arrest in human breast cancer cells. FEBS Lett..

[80] Laezza C., Pisanti S., Malfitano A.M., Bifulco M. (2008). The anandamide analog, Met-F-AEA, controls human breast cancer cell migration via the RHOA/RHO kinase signaling pathway. Endocr. Relat. Cancer..

[81] Grimaldi C., Pisanti S., Laezza C., Malfitano AM., Santoro A., Vitale M., Caruso M.G., Notarnicola M., Iacuzzo I., Portella G. (2006). Anandamide inhibits adhesion and migration of breast cancer cells. Exp. Cell Res..

[82] Mohammadpour F., Ostad S.N., Aliebrahimi S., Daman Z. (2017). Anti-invasion Effects of Cannabinoids Agonist and Antagonist on Human Breast Cancer Stem Cells. Iran. J. Pharm. Res..

[83] Pisanti S., Borselli C., Oliviero O., Laezza C., Gazzerro P., Bifulco M. (2007). Antiangiogenic activity of the endocannabinoid anandamide: Correlation to its tumor-suppressor efficacy. J. Cell. Physiol..

[84] Blasco-Benito S., Moreno E., Seijo-Vila M., Tundidor I., Andradas C., Caffarel M.M., Caro-Villalobos M., Urigüen L., Diez-Alarcia R., Moreno-Bueno G. (2019). Therapeutic targeting of HER2-CB2R heteromers in HER2-positive breast cancer. Proc. Natl. Acad. Sci. USA.

[85] Sarfaraz S., Afaq F., Adhami V.M., Mukhtar H. (2005). Cannabinoid receptor as a novel target for the treatment of prostate cancer. Cancer Res..

[86] Mimeault M., Pommery N., Wattez N., Bailly C., Hénichart J.P. (2003). Anti-proliferative and apoptotic effects of anandamide in human prostatic cancer cell lines: Implication of epidermal growth factor receptor down-regulation and ceramide production. Prostate.

[87] Nithipatikom K., Endsley MP., Isbell M.A., Falck J.R., Iwamoto Y., Hillard CJ., Campbell W.B. (2004). 2-arachidonoylglycerol: A novel inhibitor of androgen-independent prostate cancer cell invasion. Cancer Res..

[88] Nithipatikom K., Gomez-Granados A.D., Tang A.T., Pfeiffer A.W., Williams C.L., Campbell W.B. (2012). Cannabinoid receptor type 1 (CB1) activation inhibits small GTPase RhoA activity and regulates motility of prostate carcinoma cells. Endocrinology.

[89] Morell C., Bort A., Vara D., Ramos-Torres A., Rodríguez-Henche N., Díaz-Laviada I. (2016). The cannabinoid WIN 55,212-2 prevents neuroendocrine differentiation of LNCaP prostate cancer cells. Prostate Cancer Prostatic Dis..

[90] Orellana-Serradell O., Poblete C.E., Sanchez C., Castellón E.A., Gallegos I., Huidobro C., Llanos MN., Contreras H.R. (2015). Proapoptotic effect of endocannabinoids in prostate cancer cells. Oncol. Rep..

[91] Sharma M., Hudson J., Adomat H., Guns E., Cox M. (2014). In vitro anticancer activity of plant-derived cannabidiol on prostate cancer cell lines. Pharmacol. Pharm..

[92] De Petrocellis L., Ligresti A., Schiano Moriello A., Iappelli M., Verde R., Stott C.G., Cristino L., Orlando P., Di Marzo V. (2013). Non-THC cannabinoids inhibit prostate carcinoma growth in vitro and in vivo: Pro-apoptotic effects and underlying mechanisms. Br. J. Pharmacol..

[93] Gandhi S., Vasisth G., Kapoor A. (2017). Systematic review of the potential role of cannabinoids as antiproliferative agents for urological cancers. Can. Urol. Assoc. J..

[94] Bermúdez-Silva F.J., Suárez J., Baixeras E., Cobo N., Bautista D., Cuesta-Muñoz A.L., Fuentes E., Juan-Pico P., Castro M.J., Milman G. (2008). Presence of functional cannabinoid receptors in human endocrine pancreas. Diabetologia.

[95] Gonzalez-Mariscal I., Krzysik-Walker S.M., Doyle M.E., Liu Q.R., Cimbro R., Calyo S.S.C., Ghosh S., Cieśla L., Moaddel R., Carlson O.D. (2016). Human CB1 receptor isoforms, present in hepatocytes and B-cells, are involved in regulating metabolism. Sci. Rep..

[96] Kim W., Doyle M.E., Liu Z., Lao Q., Shin Y.K., Carlson O.D., Kim H.S., Thomas S., Napora J.K., Lee E.K. (2011). Cannabinoids inhibit insulin receptor signaling in pancreatic beta-cells. Diabetes.

[97] Liu J., Zhou L., Xiong K., Godlewski G., Mukhopadhyay B., Tam J., Yin S., Gao P., Shan X., Pickel J. (2012). Hepatic cannabinoid receptor-1 mediates diet-induced insulin resistance via inhibition of insulin signaling and clearance in mice. Gastroenterology.

[98] Linari G., Agostini S., Amadoro G., Ciotti M.T., Florenzano F., Improta G., Petrella C., Severini C., Broccardo M. (2009). Involvement of cannabinoid CB1- and CB2-receptors in the modulation of exocrine pancreatic secretion. Pharmacol. Res..

[99] Carracedo A., Gironella M., Lorente M., Garcia S., Guzmán M., Velasco G., Iovanna J.L. (2006). Cannabinoids induce apoptosis of pancreatic tumor cells via endoplasmic reticulum stress-related genes. Cancer Res..

[100] Dando I., Donadelli M., Costanzo C., Dalla Pozza E., D’Alessandro A., Zolla L., Palmieri M. (2013). Cannabinoids inhibit energetic metabolism and induce AMPK-dependent autophagy in pancreatic cancer cells. Cell Death Dis..

[101] Donadelli M., Dando I., Zaniboni T., Costanzo C., Dalla Pozza E., Scupoli M.T., Scarpa A., Zappavigna S., Marra M., Abbruzzese A. (2011). Gemcitabine/cannabinoid combination triggers autophagy in pancreatic cancer cells through a ROS-mediated mechanism. Cell Death Dis..

[102] Qiu C., Yang L., Wang B., Cui L., Li C., Zhuo Y., Zhang L., Zhang S., Zhang Q., Wang X. (2019). The role of 2-arachidonoylglycerol in the regulation of the tumor-immune microenvironment in murine models of pancreatic cancer. Biomed. Pharmacother..

[103] Fogli S., Nieri P., Chicca A., Adinolfi B., Mariotti V., Iacopetti P., Breschi M.C., Pellegrini S. (2006). Cannabinoid derivatives induce cell death in pancreatic MIA PaCa-2 cells via a receptor-independent mechanism. FEBS Lett..

[104] Lakiotaki E., Giaginis C., Tolia M., Alexandrou P., Delladetsima I., Giannopoulou I., Kyrgias G., Patsouris E., Theocharis S. (2015). Clinical Significance of Cannabinoid Receptors CB1 and CB2 Expression in Human Malignant and Benign Thyroid Lesions. Biomed. Res. Int..

[105] Cozzolino R., Calì G., Bifulco M., Laccetti P. (2010). A metabolically stable analogue of anandamide, Met-F-AEA, inhibits human thyroid carcinoma cell lines by activation of apoptosis. Invest. New Drugs..

[106] Shi Y., Zou M., Baitei E.Y., Alzahrani A.S., Parhar R.S., Al-Makhalafi Z., Al-Mohanna F.A. (2008). Cannabinoid 2 receptor induction by IL-12 and its potential as a therapeutic target for the treatment of anaplastic thyroid carcinoma. Cancer Gene Ther..

[107] Kushchayeva Y., Jensen K., Burman K.D., Vasko V. (2014). Repositioning therapy for thyroid cancer: New insights on established medications. Endocr. Relat. Cancer.

[108] López-Valero I., Saiz-Ladera C., Torres S., Hernández-Tiedra S., García-Taboada E., Rodríguez-Fornés F., Barba M., Dávila D., Salvador-Tormo N., Guzmán M. (2018). Targeting Glioma Initiating Cells with A combined therapy of cannabinoids and temozolomide. Biochem. Pharmacol..

[109] Chen D.J., Gao M., Gao F.F., Su Q.X., Wu J. (2017). Brain cannabinoid receptor 2: Expression, function and modulation. Acta Pharmacol. Sin..

[110] Dumitru C.A., Sandalcioglu I.E., Karsak M. (2018). Cannabinoids in Glioblastoma Therapy: New Applications for Old Drugs. Front. Mol. Neurosci..

[111] Rocha F.C., Dos Santos Júnior J.G., Stefano S.C., da Silveira D.X. (2014). Systematic review of the literature on clinical and experimental trials on the antitumor effects of cannabinoids in gliomas. J. Neurooncol..

[112] Massi P., Valenti M., Solinas M., Parolaro D. (2010). Molecular mechanisms involved in the antitumor activity of cannabinoids on gliomas: Role for oxidative stress. Cancers.

[113] Marcu J.P., Christian R.T., Lau D., Zielinski A.J., Horowitz M.P., Lee J., Pakdel A., Allison J., Limbad C., Moore D.H. (2010). Cannabidiol enhances the inhibitory effects of delta9-tetrahydrocannabinol on human glioblastoma cell proliferation and survival. Mol. Cancer Ther..

[114] Scott K.A., Dennis J.L., Dalgleish A.G., Liu W.M. (2015). Inhibiting Heat Shock Proteins Can Potentiate the Cytotoxic Effect of Cannabidiol in Human Glioma Cells. Anticancer Res..

[115] Galanti G., Fisher T., Kventsel I., Shoham J., Gallily R., Mechoulam R., Lavie G., Amariglio N., Rechavi G., Toren A. (2008). Delta 9-tetrahydrocannabinol inhibits cell cycle progression by downregulation of E2F1 in human glioblastoma multiforme cells. Acta Oncol..

[116] Blázquez C., Casanova M.L., Planas A., Gómez Del Pulgar T., Villanueva C., Fernández-Aceñero M.J., Aragonés J., Huffman J.W., Jorcano J.L., Guzmán M. (2003). Inhibition of tumor angiogenesis by cannabinoids. FASEB J..

[117] Blázquez C., González-Feria L., Alvarez L., Haro A., Casanova M.L., Guzmán M. (2004). Cannabinoids inhibit the vascular endothelial growth factor pathway in gliomas. Cancer Res..

[118] Solinas M., Massi P., Cantelmo A.R., Cattaneo M.G., Cammarota R., Bartolini D., Cinquina V., Valenti M., Vicentini L.M., Noonan D.M. (2012). Cannabidiol inhibits angiogenesis by multiple mechanisms. Br. J. Pharmacol..

[119] Ciaglia E., Torelli G., Pisanti S., Picardi P., D’Alessandro A., Laezza C., Malfitano A.M., Fiore D., Pagano Zottola A.C., Proto M.C. (2015). Cannabinoid receptor CB1 regulates STAT3 activity and its expression dictates the responsiveness to SR141716 treatment in human glioma patients’ cells. Oncotarget.

[120] Manini I., Caponnetto F., Bartolini A., Ius T., Mariuzzi L., Di Loreto C., Beltrami A.P., Cesselli D. (2018). Role of Microenvironment in Glioma Invasion: What We Learned from In Vitro Models. Int. J. Mol. Sci..

[121] Soroceanu L., Murase R., Limbad C., Singer E., Allison J., Adrados I., Kawamura R., Pakdel A., Fukuyo Y., Nguyen D. (2013). Id-1 is a key transcriptional regulator of glioblastoma aggressiveness and a novel therapeutic target. Cancer Res..

[122] Solinas M., Massi P., Cinquina V., Valenti M., Bolognini D., Gariboldi M., Monti E., Rubino T., Parolaro D. (2013). Cannabidiol, a non-psychoactive cannabinoid compound, inhibits proliferation and invasion in U87-MG and T98G glioma cells through a multitarget effect. PLoS ONE.

[123] Blázquez C., Salazar M., Carracedo A., Lorente M., Egia A., González-Feria L., Haro A., Velasco G., Guzmán M. (2008). Cannabinoids inhibit glioma cell invasion by down-regulating matrix metalloproteinase-2 expression. Cancer Res..

[124] Liebelt B.D., Shingu T., Zhou X., Ren J., Shin S.A., Hu J. (2016). Glioma Stem Cells: Signaling, Microenvironment, and Therapy. Stem Cells Int..

[125] Aguado T., Carracedo A., Julien B., Velasco G., Milman G., Mechoulam R., Alvarez L., Guzmán M., Galve-Roperh I. (2007). Cannabinoids induce glioma stem-like cell differentiation and inhibit gliomagenesis. J. Biol. Chem..

[126] Nabissi M., Morelli M.B., Santoni M., Santoni G. (2013). Triggering of the TRPV2 channel by cannabidiol sensitizes glioblastoma cells to cytotoxic chemotherapeutic agents. Carcinogenesis.

[127] Singer E., Judkins J., Salomonis N., Matlaf L., Soteropoulos P., McAllister S., Soroceanu L. (2015). Reactive oxygen species-mediated therapeutic response and resistance in glioblastoma. Cell Death Dis..

[128] Schultz S., Beyer M. (2017). GW Pharmaceuticals Achieves Positive Results in Phase 2 Proof of Concept Study in Glioma. http://ir.gwpharm.com/static-files/cde942fe-555c-4b2f-9cc9-f34d24c7ad27.

[129] Schultz S. GW Pharmaceuticals Plc Investor Presentation—February 2018. http://ir.gwpharm.com/static-files/e7afbad8-ab2c-4c8a-8e21-b9d3a7d36c70.

[130] Forte I.M., Indovina P., Iannuzzi C.A., Cirillo D., Di Marzo D., Barone D., Capone F., Pentimalli F., Giordano A. (2019). Targeted therapy based on p53 reactivation reduces both glioblastoma cell growth and resistance to temozolomide. Int. J. Oncol..

[131] Aparicio-Blanco J., Romero I.A., Male D.K., Slowing K., García-García L., Torres-Suárez A.I. (2019). Cannabidiol Enhances the Passage of Lipid Nanocapsules across the Blood-Brain Barrier Both In Vitro and In Vivo. Mol. Pharm..

