# Supplementary material for: The Endocannabinoid System: A Target for Cancer Treatment
Source: Int J Mol Sci. 2020 Jan 23;21(3):747. doi: 10.3390/ijms21030747 (PMC7037210; doi:10.3390/ijms21030747)
Supplement: Supplementary file 1 [file ijms-21-00747-s001.pdf]

Table S1. Anticancer effects of cannabinoids with relative references

Cannabinoid	Effects	References

**Anandamide (AEA)**	Lung cancer:<br>• Inhibition of lung cancer cells spreading<br>Gastrointestinal cancer:<br>• Reduction of cell volume and density; induction of G0/G1 cell cycle arrest and apoptosis (Gastric)<br>• Reduction of tumor growth *in vivo*; downregulation of angiogenic factors VEGF-C, VEGF-R2, and VEGF–R3 *in vivo* (Cholangiocarcinoma)<br>• Tumor suppressive effect via GPR55 depending on JNK activation *in vitro* and *in vivo*(Cholangiocarcinoma)<br>Breast cancer:<br>• Modulation cAMP/protein kinase A and MAPK pathway<br>• Inhibition cell cycle progression G1/S transition<br>Prostate cancer:<br>• Decrease the proliferative action of EGF and cycle cellular arrest in the G1 phase<br>• Increase apoptosis and necrosis	• Winkler et al. [17]<br>• Ortega et al. [42]<br>• DeMorrow et al. [43]<br>• DeMorrow et al. [43] Huang et al. [44]<br>• Kiskovà et al. [74]<br>• Mimeault et al. [86]<br>• Sarfaraz et al. [85]
**R(+)-methanandamide (Met-AEA o AM-356)**	Gastrointestinal cancers:<br>• G0/G1 cell cycle arrest and necrosis induction (Gastric)	• Ortega et al. [42]
**2-methyl-2′-F-anandamide (Met-F-AEA)**	Lung cancer:<br>• In combination with the FAAH inhibitor URB597 caused G0/G1 cell cycle arrest mediated apoptosis<br>• Reduced metastasis inhibiting migratory structures formation as well as MMPs secretion<br>Gastrointestinal cancers:<br>• Increase of AEA availability; decrease of proliferation rate due to CB1 up-regulation through the transcriptional activation of CNR1 promoter(Colorectal)<br>Breast cancer:<br>• Antipoliferative activity<br>• Inhibition of the EMT<br>Thyroid cancer:<br>• Increase apoptosis via activation of p53 signalling and expression of p21	• Ravi et al. [66]<br>• Ravi ey al. [71]<br>• Proto et al.[45]<br>• Laezza et al.,[79]<br>• Laezza et al., [80]<br>• Grimaldi et al [81]<br>• Cozzolino et al. [105]
**arachidonyl-2-chloroethylamide (ACEA)**	Lung cancer:<br>• Inhibition of angiogenic and lymphangiogenic factors release<br>Breast cancer:<br>• Decrease the invasive potential of breast cancer stem cells<br>Pancreatic cancer:<br>• Induction of ROS-mediated autophagy via activation of AMPK and inhibition of energetic metabolism	• Staiano et al. [63]<br>• Elbaz et al. [76]<br>• Dando et al. [100]

	• Inhibition of glycolysis via decreasing of the glycolytic enzymes, GAPDH and PKM2<br>• Increase of the anticancer potential in combination with gemcitabine	• Donadelli et al.[101]
**Δ9-tetrahydrocannabinol (THC o Δ9-THC)**	<u>Brain Cancer :</u><br>• THC reduce tumor grothw in orthotopic and subcutaneous animal models of glioma<br>• Increase in formation of ROS linked with apoptosis<br>• THC and /or CBD induce a cell cycle arrest<br>• THC could reduce pro-angiogenic VEGF levels in two patients with recurrent GBM<br>• THC was able to down regulate TIMP-1 and MMP-2<br>• clinical trial NCT01812603 of THC:CBD in combination with dose-intense TMZ<br><u>Lung cancer</u>:<br>• Inhibition of tumor growth *in vivo* and *in vitro*<br>• Reduction of signalling molecules(FAK, ERK1/2 and AKT) involved in survival and ECM remodelling<br><u>Gastrointestinal cancers</u>:<br>• Induction of apotosis and downregulation of PI3K/Akt pathway (Colorectal)<br>• Inhibition of tumor growth *in vitro* and *in vivo* associated to increased ceramide, ER-stress, PPAR-γ activity and autophagy (Hepatocellular)<br><u>Pancreatic cancer:</u><br>• Induction of apoptosis via stimulation of the *de novo* ceramide synthesis and consequent up-regulation of ER stress-related genes *p8*, *atf-4* and *trb3*.	 • Rocha et al. [111]<br>• Marcu et al. [113]<br>• Marcu et al., [113]<br>• Blàzquez et al. [117]<br>• Blàzquez et al., [123]<br>• Schultz and Beyer, [128,129],<br>• Munson et al., [65]<br>• Preet et al., [67]<br><br>• Greenhough et al. [52]<br>• Vara et al. [53]<br><br><br>• Carracedo et al. [99]
**Cannabidiol (CBD)**	<u>Brain cancer:</u><br>• CBDreduce tumor grothw in orthotopic and xenograft animal models of Glioma<br>• Increase of ROS and upregulation of heat-shock protein (HSP) super family<br>• Induce endothelial cell cytostasis, , inhibited endothelial cell migration and angiogenesis in vivo<br>• Anti-invasive effect in GBM cell lines with inhibition of Id-1 expression<br>• Treatment with CBD inducing autophagy and abrogating the chemoresistance of GSCs at BCNU therapy<br><u>Lung cancer</u>:<br>• PPARγ-dependent apoptosis; decrease of cellular migration associated to ICAM1 and TIMP1 induction<br>• Decrease of invasiveness associated to PAI1 downregulation<br><u>Gastrointestinal cancer</u>:<br>• Decrease of cell proliferation, increase of endocannabinoid levels and chemoprotective effect DNA from oxidative insults*in vitro*;reduction of invasion and migration*in vitro*(Colorectal)<br>• Decrease of aberrant crypt foci (ACF) formation, precancerous polyps and tumors in AOM-treated mice counteracting Akt activation induced by AOM (Colorectal)	 • Rocha et al. [111]<br>• Scott et al. [114]<br>• Solinas et al. [118]<br>• Soroceanu et al.[121]<br>• Nabissi et al. [126]<br><br><br>• Ramer et al. [5]<br><br>• Ramer et al. [70]<br><br>• Aviello et al. [49]

	• Induction of apoptosis due to ROS production by mitochondria, ER stress induction and NoxA activation (Colorectal)<br>• Anti-angiogenetic and anti-metastatic effects associated to VEGF downregulation*in vivo;* reduction IL-6 and IL-8 serum levels (Colorectal)<br><u>Breast cancer</u><br>• Inhibition of cell viability<br>• Induction of apoptosis/autophagy and ROS generation<br>• Cell cycle arrest at the G1/S transition (via CB1-R) and at the G2/M phase (via CB2-R)<br>• Invasiveness reduction via ID-1<br>• Inhibition cell migration and angiogenesis<br>• Modulation tumor microenvironment and cytokine production<br>• Increase overexpression of the TRPV2 in TNBC cells<br><u>Prostate cancer:</u><br>• Inhibits spheroid formation in cancer stem cells from LNCaP<br>• Downregulate VEGF, PSA and proinflammatory cytokines IL-6/IL.8<br>• In *vivo* reduce tumor size in LNCaP xenografted mice	• Jeong et al. [50]<br>• Honarmand et al. [51]<br>• Shivastava et al. [75]<br>• Kiskovà et al. [74]<br>• Elbaz et al. [76]<br>• Elbaz et al. [78]<br>• Sharma et al. [91]<br>• De Patrocellis et al. [92]
**Cannabinol (CBN)**	<u>Lung cancer</u>:<br>• Inhibition of tumor growth *in vivo* and *in vitro*	• Munson et al. [65]
**JWH-015**	<u>Lung cancer</u>:<br>• EMT inhibition and TAMs recruitment inhibition at the tumor site *in vivo*<br>• Inhibition of EGF-induced proliferation, migration and invasion in NSCLC cell lines and tumor growth and dissemination *in vivo*<br><u>Gastrointestinal cancers</u>:<br>• Inhibition of tumor growth in vitro and in vivo associated to increased ceramide, ER-stress, PPAR-γ activity and autophagy (Hepatocellular)	• Ravi et al. [71]<br>• Preet et al.[64]<br>• Vara et al. [53]
**JWH-133**	<u>Brain Cancer:</u><br>• Inibithion of angiogenesis of malignant gliomas after local administration of nonpsychotic cannabinoid JWH-133 to mice<br><u>Lung cancer</u>:<br>• Decrease of cellular migration after ICAM1 and TIMP1 induction<br>• Inhibition of angiogenic and lymphangiogenic factors release<br><u>Thyroid cancer</u>:<br>• Regression of thyroid tumours generated in nude mice by inoculation of the TC cells ARO/CB2	• Blàzquez et al. [116]<br>• Ramer et al. [5]<br>• Staiano et al. [63]<br>• Shi et al., [106]
**Win55,212-2**	<u>Lung cancer</u>:	

	• Inhibition of EGF-induced proliferation, migration and invasion in NSCLC cell lines and tumor growth and dissemination *in vivo*<br><u>Gastrointestinal cancer:</u><br>• Inhibition of proliferation and pro-apoptotic effect*in vitro*(Gastric)<br>• Inhibition of Akt activation and release inhibition of pro-migratory factors (MMP2, VEGF-A) *in vitro* and *in vivo* (Gastric)<br><u>Prostate cancer:</u><br>• Inhibition cells survival, growth and proliferation by inhibition of PI3K/Akt/mTOR axis	• Preet et al. [64]<br>• Xian et al. [59]<br>• Xian et al. [60]<br>• Morell et al. [89]
**AM251**	<u>Pancreatic cancer:</u><br>• Induction of cytotoxic effects via a receptor-independent mechanism in Mia PaCa2 cell line	• Fogli et al. [103]
**pyrrolo-1,5-benzoxazepine-15**	<u>Gastrointestinal cancer:</u><br>• Inhibition of proliferation and pro-apoptotic effect in CRC cell lines; synergistic interaction with 5-FU(Colorectal)	• Fiore et al. [46]
**Rimonabant (SR141716)**	<u>Brain Cancer:</u><br>• Cell proliferation arrest, induction caspase-dependent apoptosis and upregulation of the expression of NKG2D ligands<br><u>Gastrointestinal cancer:</u><br>• Induction of G2/M arrest and mitotic catastrophe *in vitro;* inhibition of ACF number *in vivo* (Colorectal)<br>• Wnt/β-catenin canonical pathway inhibition *in vitro* and *in vivo* associated to β-catenin degradation and TCF/LEF transcriptional inhibition (Colorectal)<br>• Improvement of 5-FU efficacy in CRC *in vitro* models;<br>• Decrease of CD133+/CD44+population and spheroid formation<br>• Synergistic effect with Oxaliplatin in CRC models	• Ciaglia et al. [119]<br>• Santoro et al. [55]<br>• Proto et al. [56]<br>• Fiore et al. [57]<br>• Gazzerro et al. [58]
**URB597**	<u>Lung cancer:</u><br>• Inhibition of lung cancer cells spreading<br>• Enforced the effect Met-F-AEA in inhibiting EGFR phosphorylation and its downstream signal transduction pathways<br><u>Gastrointestinal cancer</u>:<br>• Reduction of CRC cell lines proliferation	• Winkler et al., 2016 [17]<br>• Ravi et al., 2014 [66]<br>• Proto et al., 2012 [45]

**GW405833**<br>**(GW)**	<u>Pancreatic cancer:</u><br>• Induction of ROS-mediated autophagy via activation of AMPK and inhibition of energetic metabolism<br>• Inhibition of glycolysis via decreasing of the glycolytic enzymes, GAPDH and PKM2<br>• Increase of the anticancer potential in combination with gemcitabine	• Dando et al., 2013 [100]<br>• Donadelli et al., 2011 [101]
**2-Arachidonoylglycerol**<br>**2-AG**	<u>Prostate cancer:</u><br>• Inactivation of protein kinase A and inhibition of the invasive ability of the cells<br><u>Pancreatic cancer:</u><br>• Inhibition of cancer cell proliferation both *in vitro* and orthotopic animal models<br>• Immunomodulatory effects in tumour microenvironment	• Nithipatikom et al., 2004 [87]<br>• Qiu et al., 2019 [102]
